# Conditional ablation of myeloid TNF increases lesion volume after experimental stroke in mice, possibly via altered ERK1/2 signaling

**DOI:** 10.1038/srep29291

**Published:** 2016-07-07

**Authors:** Bettina Hjelm Clausen, Matilda Degn, Mithula Sivasaravanaparan, Torben Fogtmann, Maria Gammelstrup Andersen, Michelle D. Trojanowsky, Han Gao, Svend Hvidsten, Christina Baun, Tomas Deierborg, Bente Finsen, Bjarne Winther Kristensen, Sara Thornby Bak, Morten Meyer, Jae Lee, Sergei A. Nedospasov, Roberta Brambilla, Kate Lykke Lambertsen

**Affiliations:** 1 Department of Neurobiology Research, Institute of Molecular Medicine, University of Southern Denmark, J.B. Winsloewsvej 21st, DK-5000 Odense C, Denmark; 2 Rigshospitalet, Department of Diagnostics, Molecular Sleep Lab, Nordre Ringvej 69, DK-2600 Glostrup, Denmark; 3 Miami Project to Cure Paralysis, University os Miami Miller School of Medicine, 1095 NW 14th Terrace, Miami, FL 33136, USA; 4 Department of Nulcear Medicine, Odense University Hospital, Sdr. Boulevard 29, DK-5000 Odense C, Denmark; 5 Department of Experimental Medical Sciences, Experimental Neuroinflammation Laboratory, Lund University, Sölveg 19, 22100 Lund, Sweden; 6 Institute of Clinical Research, University of Southern Denmark, J.B. Winsloewsvej 19, DK-5000 Odense C, Denmark; 7 Department of Pathology, Odense University Hospital, Sdr. Boulevard 29, DK-5000 Odense C, Denmark; 8 Engelhardt Institute of Molecular Biology, Russian Academy of Sciences and Lomonosov Moscow State University, Vavilova Str 32, Moscow, 119991, Russia; 9 Department of Neurology, Odense University Hospital, Sdr. Boulevard 29, DK-5000 Odense C, Denmark

## Abstract

Microglia are activated following cerebral ischemia and increase their production of the neuro- and immunomodulatory cytokine tumor necrosis factor (TNF). To address the function of TNF from this cellular source in focal cerebral ischemia we used *TNF* conditional knock out mice (LysMcreTNF^fl/fl^) in which the *TNF* gene was deleted in cells of the myeloid lineage, including microglia. The deletion reduced secreted TNF levels in lipopolysaccharide-stimulated cultured primary microglia by ~93%. Furthermore, phosphorylated-ERK/ERK ratios were significantly decreased in naïve LysMcreTNF^fl/fl^ mice demonstrating altered ERK signal transduction. Micro-PET using ^18^[F]-fluorodeoxyglucose immediately after focal cerebral ischemia showed increased glucose uptake in LysMcreTNF^fl/fl^ mice, representing significant metabolic changes, that translated into increased infarct volumes at 24 hours and 5 days compared to littermates (TNFfl/fl). In naïve LysMcreTNF^fl/fl^ mice cytokine levels were low and comparable to littermates. At 6 hours, TNF producing microglia were reduced by 56% in the ischemic cortex in LysMcreTNF^fl/fl^ mice compared to littermate mice, whereas no TNF^+^ leukocytes were detected. At 24 hours, pro-inflammatory cytokine (TNF, IL-1β, IL-6, IL-5 and CXCL1) levels were significantly lower in LysMcreTNF^fl/fl^ mice, despite comparable infiltrating leukocyte populations. Our results identify microglial TNF as beneficial and neuroprotective in the acute phase and as a modulator of neuroinflammation at later time points after experimental ischemia, which may contribute to regenerative recovery.

Microglial activation constitutes a major defense mechanism against ischemic brain injury, with dual effects linking microglial activation to both neurotoxic and neuroprotective effects in the brain^1^,^2^. When activated, microglial cells become the main producers of proinflammatory cytokines, such as tumor necrosis factor (TNF), which is crucial in detemining neuronal survival and orchestrating neuroinflammation following stroke^2^,^3^,^4^,^5^. TNF exists as both transmembrane TNF (mTNF) and soluble TNF (solTNF), which is released into the extracellular space after cleavage of mTNF by the metalloproteinase TNFα converting enzyme (TACE/ADAM17)^5^. Both forms are biologically active but have distinct functions in the CNS^5^,^6^,^7^,^8^,^9^.

The effect of TNF is mediated by two cell surface receptors, TNF-R1 and TNF-R2. These receptors may feed into diverse signaling pathways according to differences in their intracellular domains. A death domain in TNF-R1, not present in TNF-R2, can lead to apoptotic cell death, although most cell types are protected due to expression of anti-apoptotic proteins. Alternative pathways of TNF-R1 signaling involve activation of the ERK1/2 MAP kinases and NF-κB activation^10^,^11^ serving neuroprotective functions. Similarly, TNFR2 receptor signaling through the NF-κB pathway is primarily associated with neuroprotection and axonal preservation^6^,^9^,^12^,^13^.

In experimental stroke, genetic ablation of solTNF, with sustained mTNF expression, is associated with reduced infarct volume, improved functional outcome and an altered pro-inflammatory environment^5^, just as the systemically administered anti-solTNF compound XPro1595 improves functional outcome after experimental stroke^3^. On the other hand, conventional TNF knock out (TNF^−/−^) mice display increased infarct volumes and worse functional outcome after experimental stroke^4^. The neuroprotective effect of TNF is suggested to be mediated through TNF-R1, findings which are in line with studies showing increased infarct volumes in TNF-R1 deficient mice^4^,^14^,^15^ and that ischemic tolerance is mediated through upregulation of TNF-R1^16^.

Given the contribution of microglia/macrophages to the pathophysiology of stroke, in the present study, we investigated the effect of TNF ablation specifically in myeloid cells on neuroprotection and functional outcome after focal cerebral ischemia. We demonstrate that LysMcreTNF^fl/fl^ mice lacking TNF in myeloid cells display phenotypical alterations in microglia, including altered ERK signaling. In addition, FDG uptake in the ischemic brain during the acute phase after ischemia (1–3 hours) is increased in LysMcreTNF^fl/fl^ mice resulting in increased infarct volumes 24 hours and 5 days after focal cerebral ischemia. The relevance of the present study was underlined by investigating glial activation, TNF and TNF receptor expression in human autopsy material from stroke patients. This may have significant implications for the development of future neuroprotective treatment strategies in the acute phase after stroke, as modulating the microglial TNF response acutely can provide a viable option to boost neuroprotection.

## Results

### Characterization of LysMcreTNF^fl/fl^ mice with conditional ablation of TNF in myeloid cells

**Figure 1:**
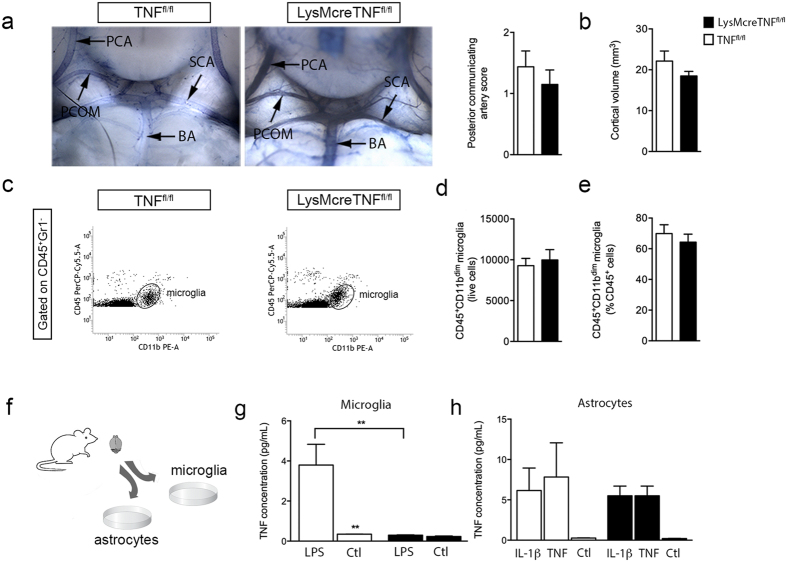
Characterization of LysMcreTNF^fl/fl^ mice. (**a**) Photomicrographs of the posterior part of the circle of Willis in TNF^fl/fl^ and LysMcreTNF^fl/fl^ mice showing similar vascular anatomy of the posterior part of the circle of Willis after carbon ink perfusion. There were no differences in the mean posterior communicating artery scores between TNF^fl/fl^ and LysMcreTNF^fl/fl^ mice based on carbon ink labeling (P = 0.42, n = 8–10/group, t-test). Abbreviations: BA, basilar artery; PCA, posterior cerebral artery; PCOM, posterior communicating artery; SCA, superior cerebellar artery. (**b**) Neocortical volumes were comparable in TNF^fl/fl^ and LysMcreTNF^fl/fl^ mice (n = 4–5/group, t-test). (**c**) Gating strategy for microglia in the cortex of naïve TNF^fl/fl^ and LysMcreTNF^fl/fl^ mice. Gate was defined to include only live cells in the further analysis. The microglial population was identified as CD11b^+^CD45^dim^ cells. (**d**,**e**) Flow cytometric analysis showed equivalent numbers (**d**) and percentages (**e**) of microglia in the cortices of naïve TNF^fl/fl^ and LysMcreTNF^fl/fl^ mice (n = 6–9/group, t-test). (**f–h**) Stimulation of TNF production in primary microglial cultures treated with LPS showed that only TNF^fl/fl^ microglia produced a robust TNF response upon stimulation, whereas TNF levels remained at baseline levels in LysMcreTNF^fl/fl^ microglia (n = 12–17 wells/experimental group, 2 independent experiments, t-test) (**g**). In contrast, primary astrocytes from both TNF^fl/fl^ and LysMcreTNF^fl/fl^ mice produced a robust TNF response upon stimulation with either IL-1β or TNF (n = 6 wells/experimental group, 2 independent experiments) (**h**). All data are presented as mean ± SEM.

In order to assess the effect of genetic ablation of TNF in myeloid cells, we first characterized LysMcreTNF^fl/fl^ mice under naïve conditions. LysMcreTNF^fl/fl^ mice were comparable to TNF^fl/fl^ mice displaying normal cerebrovascular anatomy at the level of the circle of Willis (Fig. 1a), similar cortical volumes (Fig. 1b), as well as body composition (Suppl. Table 1). Furthermore, conditional TNF ablation resulted in no abnormal locomotor function, as previously observed in conventional TNF^−/−^ mice^5^, when subjected to the rotarod and open field test (Suppl. Table 1). We found LysMcreTNF^fl/fl^ mice to have normal neuromuscular function, balance, motor coordination and spontaneous activity (Suppl. Table 1), just as anxiety-related behaviors (grooming, fecal droppings, latency to rear and center/peri-meter ratio) were not altered compared to TNF^fl/fl^ mice (Suppl. Table 1). When testing for spontanoeus alternation behavior (SAB) and working memory, we found no difference in SAB % but significantly fewer Y-maze entries in LysMcreTNF^fl/fl^ mice compared to controls (Suppl. Table 1).

Since conventional TNF^−/−^ mice are known to display reduced numbers of microglia^4^, we next quantified microglia by flow cytometry using CD45 levels to distinguish CD45^dim^ microglia from infiltrating CD45^high^ leukocytes (Fig. 1c–e). We found comparable microglial numbers (Fig. 1d) and percentages (Fig. 1e) between TNF^fl/fl^ and LysMcreTNF^fl/fl^ mice. However, mean fluorescence intensity (MFI) revealed higher CD11b (Suppl. Fig. 1a) and lower CD45 (Suppl. Fig. 1b) expression levels on microglia in naïve LysMcreTNF^fl/fl^ compared to TNF^fl/fl^ mice. Analysis of whole blood showed comparable numbers of monocytes and T cells, but fewer Gr1^+^ granulocytes in LysMcreTNF^fl/fl^ compared to TNF^fl/fl^ mice (Suppl. Fig. 1c,d). In the spleen, monocytes, granulocytes and T cells were comparable between the two genotypes (Suppl. Fig. 1c,d).

We addressed recombination efficiency in microglial cells by crossing MLys1^cre^ mice with Rosa26-tdTomato reporter mice and quantified the number of cortical tdTomato^+^ cells in naïve conditions by cell counting and flow cytometry (Suppl. Fig. 2). Under naïve conditions, we found 12% (cell counting) to 20% (flow cytometry) of all CD11b^+^ microglial cells to be tdTomato^+^. No tdTomato^+^ cells co-labelled with GFAP or Olig2, but a small proportion of neurons were tdTomato^+^ (5%). Since microglia increase LysM expression under ischemic and inflammatory conditions^17^,^18^,^19^, we next tested the cre-mediated reporter gene activation under inflammatory conditions using cultured microglia harvested from TNF^fl/fl^ and LysMcreTNF^fl/fl^ mice (Fig. 1f,g). Following LPS-stimulation, we found that microglia from LysMcreTNF^fl/fl^ mice showed a ∼93% decrease in TNF production as compared to microglia from TNF^fl/fl^ mice (Fig. 1g). Astrocytes stimulated with either IL-1β or TNF secreted comparable amounts of TNF, with no difference between TNF^fl/fl^ and LysMcreTNF^fl/fl^ mice (Fig. 1h), collectively demonstrating a successful TNF ablation in activated microglia from LysMcreTNF^fl/fl^ mice.

Altogether, these data demonstrate a successful TNF ablation in activated microglia and support the use of LysMcreTNF^fl/fl^ mice as a suitable model to study microglial/myeloid TNF function *in vivo*.

### Conditional ablation of myeloid TNF increases infarct volume after focal cerebral ischemia

**Figure 2:**
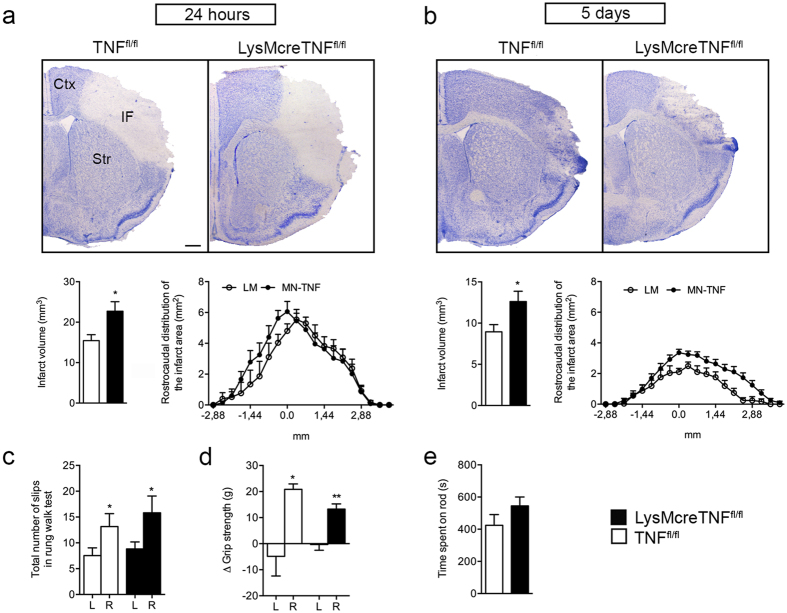
Infarct volumes are increased in LysMcreTNF^fl/fl^ mice. (**a**,**b**) Toluidine blue staining of brain sections from TNF^fl/fl^ and LysMcreTNF^fl/fl^ mice 24 hours (**a**) and 5 days (**b**) after focal cerebral ischemia. Abbreviations: Ctx, cortex; IF, infarct; Str, striatum. Scale bar = 500 μm. Estimation of cortical infarct volumes showed that LysMcreTNF^fl/fl^ mice developed significantly larger infarcts compared with TNF^fl/fl^ both at 24 hours (**a)**, (left graph) and 5 days (**b)**, (left graph) (n = 8–12/group and n = 15–25, respectively, t-test). Analysis of the rostrocaudal distribution of the infarct areas 24 hours (**a**), (right graph) and 5 days (**b)**, (right graph) after focal cerebral ischemia showed that TNF^fl/fl^ and LysMcreTNF^fl/fl^ mice displayed similar rostrocaudal distribution of the infarcts. (**c**) Rung walk analysis at day 2 showed that both TNF^fl/fl^ and LysMcreTNF^fl/fl^ mice presented with significantly more total slips on their right limbs compared to their left limbs (n = 6–13/group, paired t-test). (**d**) Grip strength analysis at day 5 showed front paw asymmetry (Δ grip strength) in both TNF^fl/fl^ and LysMcreTNF^fl/fl^ mice (n = 5–6/group, paired t-test). (**e**) Rotarod performance at day 5 was comparable between TNFfl/fl and LysMcreTNFfl/fl mice (n = 10–14/group). *P ≤ 0.05, **P ≤ 0.01. All data are presented as mean ± SEM.

The effect of myeloid TNF ablation in cerebral ischemia was assessed by comparing infarct volumes in TNF^fl/fl^ to LysMcreTNF^fl/fl^ mice after pMCAO (Fig. 2a,b). In LysMcreTNF^fl/fl^ mice infarct volumes were significantly larger than in TNF^fl/fl^ mice at 24 hours (30%) (Fig. 2a) and at 5 days (45%) (Fig. 2b) after pMCAO. Histological analysis showed that infarcts were evenly distributed along the rostrocaudal axis in both genotypes (Fig. 2a,b).

To assess functional outcome, we subjected the mice to a battery of behavioral tests, but found no locomotor or sensory-motor differences between TNF^fl/fl^ and LysMcreTNF^fl/fl^ mice 2 and 5 days after pMCAO (Suppl. Table 2). Rung walk analysis of both front and hinds paws (total slips) on day 2 after pMCAO showed clear asymmetry between the left (L) and right (R) side (Fig. 2c), as did grip strength of the front paws 5 days after pMCAO (Fig. 2d) in both TNF^fl/fl^ and LysMcreTNF^fl/fl^ mice. Both groups of mice performed similarly on the rotarod 5 days after pMCAO (Fig. 2e). In the open field test 2 days after pMCAO, we found no difference between TNF^fl/fl^ and LysMcreTNF^fl/fl^ mice in total distance traveled, speed, zone changes and number of rearings (Suppl. Table 2). Also no change in anxiety-related behaviors, such as center/perimeter ratio, latency to rear and number of droppings were found, except for grooming behavior, which was significantly increased in TNF^fl/fl^ mice after pMCAO (Suppl. Table 2). The lack of differences in functional outcome most likely reflects the large cortical infarcts in both genotypes.

### FDG uptake is increased in the acute phase after pMCAO in LysMcreTNF^fl/fl^ compared to TNF^fl/fl^ mice

**Figure 3:**
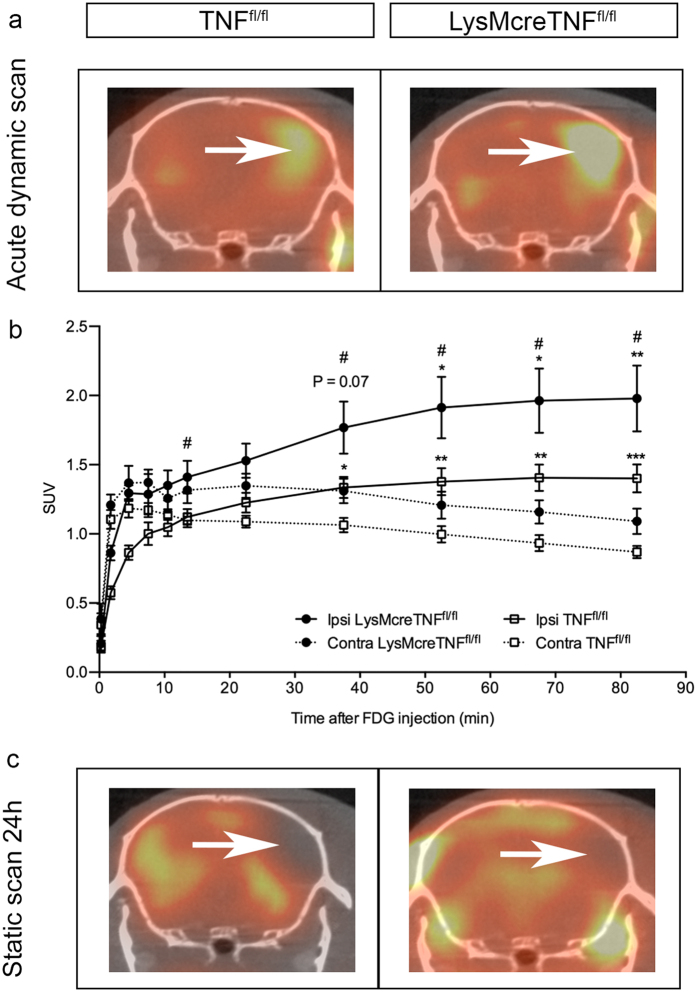
Dynamic PET analysis of LysMcreTNF^fl/fl^ and TNF^fl/fl^ mice in the acute phase after pMCAO. (**a**) Coregistered computed tomographic (CT) images and position emission tomographic (PET) images from TNF^fl/fl^ and LysMcreTNF^fl/fl^ mice with [^18^]F-2-fluoro-2-deoxy-D-glucose (FDG) injected 30 min after pMCAO. (**b**) Dynamic uptake curves from a cortical region in the contralateral hemisphere (dashed lines) and ischemic region (solid lines). The ischemic region was defined from the last PET frame showing elevated FDG uptake. FDG uptake in the ischemic region at 67.5–82.5 min was significantly higher than that in the contralateral hemisphere in LysMcreTNF^fl/fl^ mice and at 37.5–85.5 min in TNF^fl/fl^ mice (*P ≤ 0.05; **P ≤ 0.01; ***P ≤ 0.001). FDG uptake was also significantly increased at 37.5–85.5 min in the ipsilateral hemisphere in LysMcreTNF^fl/fl^ mice compared to the ipsilateral hemisphere in TNF^fl/fl^ mice (^#^P ≤ 0.05) (n = 4–5/group, Two-way ANOVA followed by Bonferroni’s corrected multiple t-test). (**c**) Coregistered CT images and PET images from LysMcreTNF^fl/fl^ and TNF^fl/fl^ mice with [^18^]F-FDG injected at 24 hours after pMCAO. Arrows indicate the ischemic infarct. SUV, standard uptake value. All data are presented as mean ± SEM.

To assess ischemia-dependent changes in glucose metabolism immediately after pMCAO (1–3 hours), we performed ^18^[F]-FDG-PET imaging (Fig. 3a). Dynamic scans revealed an early (<10 min) reduction in FDG uptake in the ipsilateral cortex compared to the contralateral in both genotypes, most likely as a result of ceased blood flow to the lesion site (Fig. 3b). Ten to 15 min after injection, FDG uptake switched and was increased in the ipsilateral side compared to the contralateral side in both TNF^fl/fl^ and LysMcreTNF^fl/fl^ mice. However, beginning approximately 35 min post-injection, ipsilateral FDG uptake was significantly higher in the ipsilateral hemisphere in LysMcreTNF^fl/fl^ compared to TNF^fl/fl^ controls (Fig. 3b), demonstrating an increased glucose demand in the ipsilateral hemisphere after pMCAO. This is consistent with the presence of larger infarcts in LysMcreTNF^fl/fl^ mice where cells increased glucose uptake after stroke onset.

VOI-based analysis of ^18^[F]-FDG-PET imaging comparing the normalized rate constants (K_i_, influx rate; K_1_, rate blood to tissue; k_2_, rate tissue to blood; k_3_, binding rate) revealed significant differences between TNF^fl/fl^ and LysMcreTNF^fl/fl^ mice (Table 1). The increase in K_i_ corresponded to a 37% elevation in glucose uptake in LysMcreTNF^fl/fl^ compared to TNF^fl/fl^ mice. A second scan 24 hours later revealed a lower ipsilateral FDG uptake in both groups (Fig. 3c) due to loss of cells in the ischemic region. However, there was no significant difference in the uptake pattern 24 hours after pMCAO, as demonstrated by previous findings of a fully mature infarct at this time point^20^.

### Ablation of myeloid TNF alters MAPK protein expression in naïve conditions but not after pMCAO

**Figure 4:**
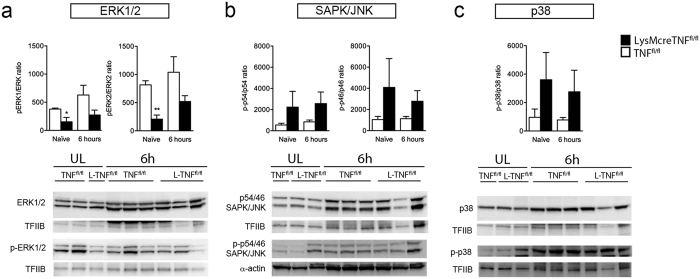
MAPK activation after pMCAO. (**a–c**) Quantification of ERK1/2 and p-ERK1/2 (**a**), JNK/SAPK and p-JNK/SAPK (**b**), p38 and p-p38 (**c**) protein expression in brain tissue of naïve TNF^fl/fl^ and LysMcreTNF^fl/fl^ mice and 6 hours after pMCAO. Data are normalized to transcription factor II B (TFIIB) or α-actin protein expression. *P ≤ 0.05; **P ≤ 0.01. Abbreviations: L-TNF^fl/fl^, LysMcreTNF^fl/fl^; UL, unlesioned. Representative experiments are shown. Results, expressed as percentage of naïve TNF^fl/fl^ mice, are mean ± SEM of three mice per group in 2–4 independent setups.

Since TNF-dependent MAPK signaling has been associated with neuroprotection under ischemic conditions^21^, we evaluated whether the increase of infarct volumes in LysMcreTNF^fl/fl^ mice could be associated with alterations in MAPK signaling. Interestingly, we found pERK1/ERK1 and pERK2/ERK2 ratios to be significantly decreased in LysMcreTNF^fl/fl^ mice compared to TNF^fl/fl^ mice under naïve conditions (60% and 75%, respectively) (Fig. 4a), suggesting that ERK signaling is intrinsically downregulated in LysMcreTNF^fl/fl^ mice, which could impair ERK-dependent neuroprotective signaling acutely (within the first 6 hours) after pMCAO. Despite tendencies, pERK1/ERK1 and pERK2/Erk2 ratios were not different between TNF^fl/fl^ and LysMcreTNF^fl/fl^ mice 6 hours after pMCAO (Fig. 4a). No differences were found in p-p54/p54, p-p46/p46 (SAPK/JNK) (Fig. 4b), or p-p38/p38 (Fig. 4c) ratios in naïve and ischemic conditions.

### Ablation of myeloid TNF reduces the ischemic-induced expression of pro-inflammatory cytokines and chemokines 24 hours after pMCAO

**Figure 5:**
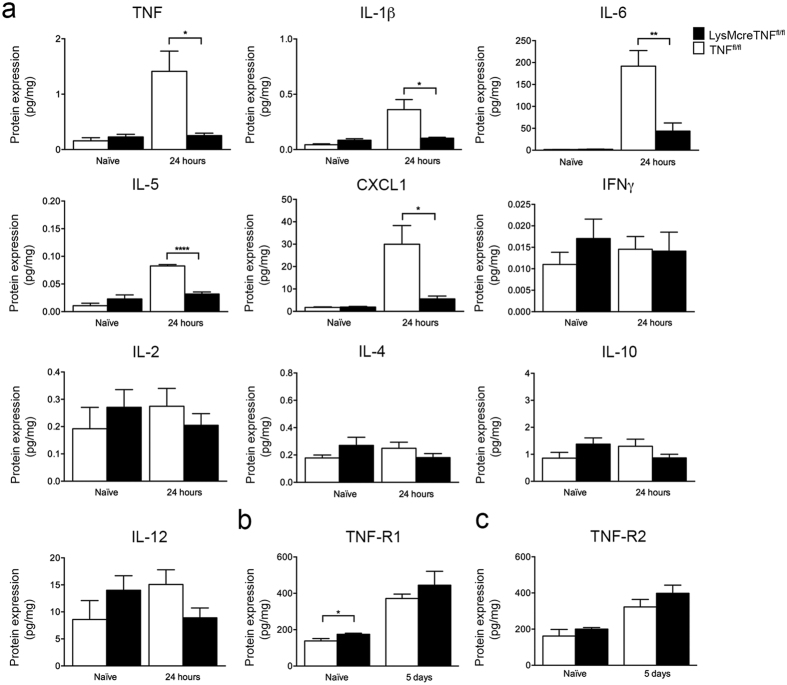
Inflammatory changes in LysMcreTNF^fl/fl^ after pMCAO. (**a**) Multiplex analysis showing comparable TNF, IL-1β, IL-6, IL-5, and CXCL1 protein levels in naïve TNF^fl/fl^ and LysMcreTNF^fl/fl^ mice but significantly increased levels 24 hours after pMCAO (*P ≤ 0.05, **P ≤ 0.01; ****P ≤ 0.0001). IFNγ, IL-2, IL-4, IL-10, and IL-12 did not change in naïve conditions or after pMCAO. (**b**) Multiplex analysis showing significanly increased TNF-R1 levels in naïve LysMcreTNF^fl/fl^ compared to TNF^fl/fl^ mice but comparable levels at 5 days after pMCAO (*P ≤ 0.05). (**c**) TNF-R2 levels were comparable in naïve conditions and 5 days after pMCAO. Results, expressed as mean ± SEM (n = 4–5/group, t-test).

To assess changes in the inflammatory response between TNF^fl/fl^ and LysMcreTNF^fl/fl^ mice, we assessed cytokine, chemokine, and TNFR protein expression in naïve conditions and after pMCAO by multiplex analysis (Fig. 5a–c and Suppl. Table 3). No differences in protein expression were found in naïve conditions except for TNFR1, which was significantly increased in LysMcreTNF^fl/fl^ compared to TNF^fl/fl^ mice (Fig. 5b). Similarly, 6 hours and 5 days after pMCAO no differences in protein expression were detected (Suppl. Table 3). At 24 hours after pMCAO, however, TNF, IL-1β, IL-6, IL-5 and CXCL1 were significantly reduced in LysMcreTNF^fl/fl^ mice compared to TNF^fl/fl^ controls, while IL-2, IL-4, IL-10, IFNγ or IL-12p70 did not change (Fig. 5a). Cytokine levels were also measured in the plasma in naïve and ischemic conditions but no changes were found at any time point between TNF^fl/fl^ and LysMcreTNF^fl/fl^ mice (Suppl. Fig. 3).

### Ablation of myeloid TNF does not result in changes in peripheral myeloid cell numbers or infiltrating immune cells after pMCAO

**Figure 6:**
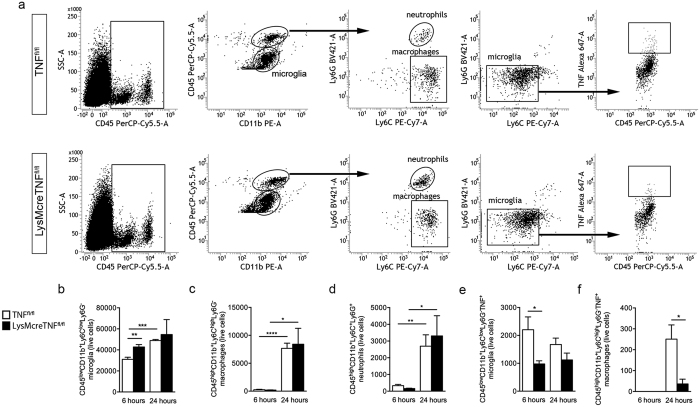
Changes in microglia and leukocyte profiles after pMCAO. (**a–f**) Flow cytometric gating strategy (**a**) and analysis of the total number of CD11b^+^CD45^dim^Ly6C^low^Ly6G^−^ microglia (**b**), CD11b^+^CD45^high^Ly6C^high^Ly6G^−^ macrophages (**c**), CD11b^+^CD45^high^Ly6C^high^Ly6G^+^ granulocytes (**d**) and TNF^+^ microglia (**e**) and TNF^+^ macrophages (**f**) in the cortex of TNF^fl/fl^ and LysMcreTNF^fl/fl^ mice 6 and 24 hours after pMCAO. *P ≤ 0.05; **P ≤ 0.01; ***P ≤ 0.001; ****P ≤ 0.0001. Results are expressed as mean ± SEM of 5 animals/group, t-test.

Based on our previous findings of increased leukocyte infiltration in conventional TNF^−/−^ mice^4^ and decreased macrophage infiltration in mice only expressing the transmembrane form of TNF (mTNF^Δ/Δ^)^5^ one day after pMCAO as well as altered microglial activation following anti-TNF treatment^3^, we investigated possible changes in microglia and infiltrating leukocytes in the cortex of TNF^fl/fl^ and LysMcreTNF^fl/fl^ mice 6 and 24 hours after pMCAO by the use of flow cytometry (Fig. 6a–d and Suppl. Fig. 4a,b). At 6 hours, we found the total number of CD11b^+^CD45^low^Ly6C^+^Ly6G^−^ microglia to be significantly increased in LysMcreTNF^fl/fl^ mice compared to TNF^fl/fl^ mice (Fig. 6b), most likely a result of increased CD11b expression in LysMcreTNF^fl/fl^ mice under naïve conditions as measured by MFI (Suppl. Fig. 1a). As expected, at 24 hours after pMCAO, the time of maximal cytokine production, in TNF^fl/fl^ and LysMcreTNF^fl/fl^ mice the percentage and number of CD11b^+^CD45^dim^ microglia were decreased in the ipsilateral cortex as compared to the contralateral (Suppl. Fig. 4a,b). However, no differences in percentage and cell number were observed between the two genotypes (Fig. 6b and Suppl. Fig. 4a,b). At 6 hours after pMCAO, the numbers of infiltrating CD11b^+^CD45^high^Ly6C^high^Ly6G^-^ macrophages (Fig. 6c) and CD11b^+^CD45^high^Ly6C^high^Ly6G^+^ granulocytes (Fig. 6d) were low and comparable between the two genotypes. By 24 hours, the percentage and number of infiltrating macrophages and granulocytes were significantly increased in the ipsilateral cortex of LysMcreTNF^fl/fl^ and TNF^fl/fl^ mice compared to the contralateral cortex, however with no difference between the two groups (Fig. 6c,d and Suppl. Fig. 4b). The number and percentage of infiltrating CD45^high^CD3^+^ T cells were minimal and did not change in the ipsilateral cortex as compared to the contralateral in either TNF^fl/fl^ or LysMcreTNF^fl/fl^ mice, nor between the two groups (Suppl. Fig. 4b).

At 6 hours, we found that the total number of TNF^+^ microglia was significantly decreased in LysMcreTNF^fl/fl^ mice as compared to TNF^fl/fl^ mice (Fig. 6e) and demonstrated an approximately 56% ablation of TNF in microglia. At 6 hours, none of the few infiltrating macrophages were found to express TNF (Fig. 6f). At 24 hours, the number of TNF^+^ macrophages was significantly decreased in LysMcreTNF^fl/fl^ compared to TNF^fl/fl^ mice (Fig. 6f) where only 0.5% of all macrophages in LysMcreTNF^fl/fl^ mice were TNF^+^ compared to 3% in TNF^fl/fl^ mice. At no time point did granulocytes express TNF. This suggests that leukocyte recruitment into the infarct area is not affected by TNF ablation in myeloid cells 6 and 24 hours after pMCAO.

By 24 hours after pMCAO the percentage of all leukocyte populations in the blood and spleen were comparable between LysMcreTNF^fl/fl^ and TNF^fl/fl^ mice (Suppl. Fig. 4d,e). In the bone marrow, CD11b^+^CD45^+^Gr1^−^ and CD45^+^CD3^+^ populations were significantly increased in LysMcreTNF^fl/fl^ mice compared to TNF^fl/fl^ mice, with a higher number and percentage of all CD45^+^ cells in LysMcreTNF^fl/fl^ mice compared to controls (Suppl. Fig. 4e).

### Localization of TNF, TNF-R1 and TNF-R2 in human stroke brains

**Figure 7:**
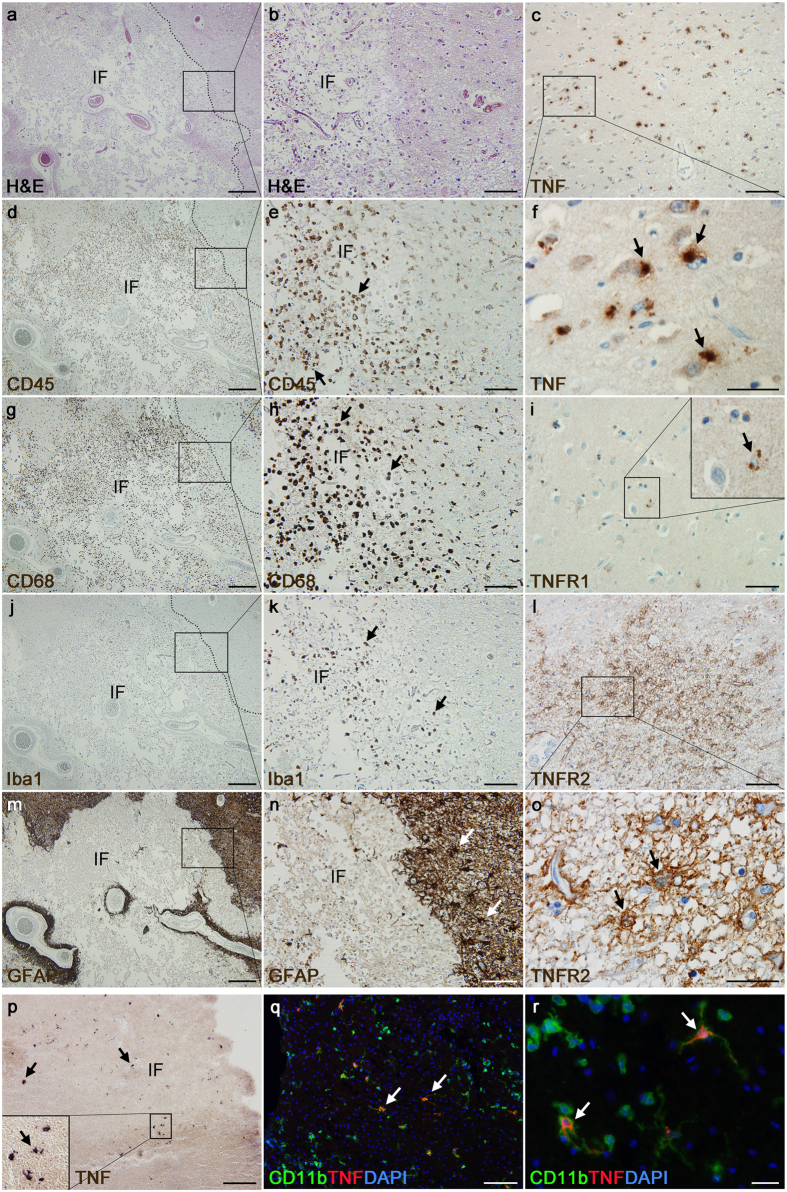
Characterization of TNF expression and localization in the brain of patients with stroke and mice with experimentally induced stroke. (**a**,**b**) Haematoxylin and eosin (HE) stain of a > 7-day old ischemic infarct placed in the left internal capsule of a stroke patient. (**c**) Diaminobenzidine (DAB) staining of TNF located within the infarct showing TNF labelling in cells morphologically resembling leukocytes/microglia. (**d**,**e**) DAB staining of CD45^+^ cells located within the infarct and peri-infarct. (**f**) High magnification of (**c**) showing TNF^+^ cells located within the infarct. (**g**,**h**) DAB staining of CD68^+^ cells located within the infarct and peri-infarct. (**i**) DAB staining of TNFR1^+^ cell located in the peri-infarct area. (**j**,**k**) DAB staining of Iba1^+^ microglia and leukocytes located within the infarct and in the peri-infarct. (**l**) DAB staining of TNFR2 located in the peri-infarct area showing TNFR2 labelling in cells with a glial morphology. (**m**,**n**) DAB staining of GFAP^+^ astrocytes located in the peri-infarct. (**o**) High magnification of (**l**) showing TNFR2^+^ cells in the peri-infarct. (**p**) Alkaline phosphatase (AP) staining of TNF located within a 24-hours old infarct and in the peri-infarct placed in the parietal cortex of a mouse showing TNF labelling in cells morphologically resembling leukocytes/microglia. (**q**) Double-immunofluorescent labelling of TNF with CD11b, demonstrating TNF expression in microglia/leukocytes. (**r**) High power magnification photomicrographs of double-fluorescent labelling of TNF with CD11b showing TNF in vesicles located both in the cytoplasm surrounding the nucleus and in the processes extending from the cells. Scale bars: (**a**,**d**,**g**,**j**,**m**) = 400 μm, (**b**,**c**,**e**,**h**,**i**,**k**,**l**,**n**) = 100 μm, (**f,o**) = 30 μm, (**p,q**) = 200 μm, (**r**) = 20 μm. Abbreviations: IF, infarct.

To address TNF signaling in the context of human stroke pathophysiology, we analyzed TNF, TNF-R1, and TNF-R2 expression in the *post-mortem* brain of two stroke cases (Suppl. Table 4 and Fig. 7a–o, shown for 1 *post-mortem* brain only). The infarcted area, identified by HE staining (Fig. 7a,b), was characterized by extensive tissue disruption and presence of cell infiltrates immunopositive for CD45 and CD68 (Fig. 7d,e,g,h). Iba1^+^ cells, mostly with macrophage morphology, were also found in high numbers within the infarct (Fig. 7j,k). GFAP^+^ astrocytes formed a demarcated glial scar and were found to be almost absent within the infarct (Fig. 7m,n). TNF^+^ cells were located primarily within the infarct and at the border between infarct and intact tissue (Fig. 7c,f). On the contrary, TNF-R1^+^ (Fig. 7i) and TNF-R2^+^ (Fig. 7l,o) cells were found in the border zone only, with TNFR2^+^ cells accumulating in a dense layer surrounding the infarct. TNFR1^+^ cells were located at the border zone and further away in the peri-infarct tissue. We also assessed TNF expression in mouse tissue 24 hours after pMCAO and found high numbers of TNF^+^CD11b^+^ cells within the infarct and peri-infarct area (Fig. 7p–r), confirming previous findings in mice^4^. No TNF^+^ astrocytes were detected (not shown).

## Discussion

Using LysMcreTNF^fl/fl^ conditional KO mice with specific ablation of TNF in myeloid cells, including microglia, we demonstrate that microglial-derived TNF contributes to TNF-mediated protective effects in experimental stroke. We and others previously demonstrated that in the CNS acutely after focal cerebral ischemia TNF is produced by microglia, not neurons or astrocytes^3^,^4^,^22^,^23^. Using chimeric mice, we also showed that microglial-derived TNF is neuroprotective whereas macrophage-derived TNF plays no role in acute infarct maturation^4^. Therefore we can conclude that worsening of the stroke pathology in LysMcreTNF^fl/fl^ mice is dependent on the lack of microglial-derived TNF.

Our findings on expression of TNF and its receptors in brain specimens from stroke patients are in line with previous reports (reviewed in^2^) and underscore this study’s translational relevance of TNF signaling in human stroke pathology, not only in experimental models.

We and others have shown that TNF^−/−^ mice exhibit a behavioral phenotype associated with increased locomotor activity and altered anxiety-related behavior, in addition to impaired spatial learning and memory^24^,^25^, while mTNF^Δ/Δ^ mice, expressing only mTNF, display a normal behavioral phenotype^5^. In this study we show that conditional ablation of myeloid TNF alone did not impair locomotor or neuromuscular function. This may depend on the relatively limited TNF ablation in microglia from LysMcreTNF^fl/fl^ mice in naïve conditions, which may not be sufficient to induce a measurable phenotype, suggesting that constitutively expressed TNF in the brain of naïve LysMcreTNF^fl/fl^ mice is sufficient for maintenance of cognitive functions under physiological conditions. Alternatively, it may be that other sources of TNF in naïve conditions are supporting normal cognitive and locomotor function.

Eventhough in LysMcreTNF^fl/fl^ mutant mice the deletion efficiency is around 85–98% in mature macrophages and near 100% in granulocytes^26^, we observed that in naïve CD11b^+^ microglia deletion efficiency was only around 20% and that recombination also occured in approximately 5% of neurons. Neuronal expression was very low, and we consistently never observed increased TNF expression in neurons in this model of experimental stroke^22^,^23^,^27^,^28^. Furthermore, microglia increase their expression of LysM in ischemic and inflammatory conditions, leading to more efficient gene ablation in LysMcre expressing mice^17^,^18^,^19^. Indeed, we first verified ablation efficiency in LPS-stimulated microglia cultures from LysMcreTNF^fl/fl^ and TNF^fl/fl^ mice by showing a marked reduction in TNF release (93%) and then by flow cytometry 6 hours after pMCAO demonstating a 56% reduction in TNF^+^ microglia in the ischemic brain. On the contrary, release of TNF was not affected *in vitro* in astrocytes, further validating efficient and selective ablation of TNF from microglial cells.

When we tested the effect of myeloid TNF deletion in experimental stroke, we found LysMcreTNF^fl/fl^ mice to have significantly increased FDG uptake acutely (>3 hours) and larger infarct volumes at 24 hours and 5 days after pMCAO, supporting our previous findings that constitutively expressed or early induced, microglial-derived TNF is neuroprotective in experimental stroke^4^. When evaluating the spatiotemporal dynamics of FDG uptake during acute pMCAO in TNF^fl/fl^ and LysMcreTNF^fl/fl^ mice, we found early microglial-derived TNF to affect FDG uptake in the ischemic tissue after pMCAO. Though assumptions of a steady state system is hampered in the acute phase after pMCAO and care should be taken not to use the kinetic model data as absolute true numbers, the findings of hyper-FDG in the ispsilateral cortex compared with the contralateral cortex within the first 2 hours after pMCAO in both strains of mice are consistent with previous findings of increased accumulation of glucose around the ischemic border^29^,^30^. This may reflect increased anaerobic glycolysis during acute cerebral ischemia to compensate the loss of ATP^29^,^30^,^31^ or alteratively that lack of microglial-derived TNF alters ongoing signaling cascades in the ischemic brain^10^,^11^. The finding of increased FDG uptake in the ipsilateral hemisphere of LysMcreTNF^fl/fl^ mice compared to TNF^fl/fl^ mice during acute ischemia supports the findings of increased infarct volumes 24 hours and 5 days after pMCAO and demonstrate that conditional ablation of TNF in microglia modifies neuronal sensitivity to ischemia. The fact that we in the present study were not able to differentiate between FDG uptake in infarct and peri-infarct areas is due to the limited resolution (1 mm) of the PET scanner as imaging spatial resolution is essential to delineate these areas^30^.

In some studies JNK/SAPK and p38 MAPK were shown to promote cell death, whereas ERK1/2 opposed cell death^32^,^33^. In the present study we found SAPK/JNK and p38 ratios not to be changed both under naïve conditions and 6 hours after pMCAO, whereas ERK1/2 ratios were significantly decreased in naïve LysMcreTNF^fl/fl^ mice, with a tendency still present 6 hours after pMCAO. These findings suggest that ERK signal transduction is altered in LysMcreTNF^fl/fl^ mice. TNF is known to activate all three MAPKs in neurons, with ERK1/2 activation mediated via MEK1/2^34^ and ERK1/2 activation is known to have a dominant-negative effect on the apoptotic signaling of death receptors, including TNF-R1^33^. However, ERK1/2 activation has been shown to primarily mediate cell protection through TNF-R1-mediated NF-κB activation^33^. We and others have previously shown that the neuroprotective effect of TNF is mediated at least in part via TNF-R1 (reviewed in^2^). Even though NF-κB activation and translocation are comparable in TNF^−/−^ and WT mice after pMCAO^4^, it is possible that ablation of microglial TNF leads to a decrease in ERK1/2 ratios and altered NF-κB activation, which ultimately results in lack of NF-κB-induced neuroprotection in LysMcreTNF^fl/fl^ mice. In support of this, Rousseau *et al*.^35^ showed that *in vitro* LPS stimulation of macrophages results in sequential activation of the TPL2-MKK1/MKK2-ERK1/2 pathway followed by secretion of TNF and that inhibition of this pathway resulted in loss of TNF production. Alternatively, we speculate that conditional ablation of TNF in microglia may have affected microglial expression of P2X_7_ receptors, as previously shown^36^. ATP, released from damaged cells as a consequence of ischemia, has been shown to act on P2X_7_ receptors on microglia and thereby activate MAPK (ERK1/2, JNK and p38), which results in the release of neuroprotective TNF^37^. Thus the balance between ERK and the JNK and p38 MAPK pathways may play an important role in neuronal cell survival or death in response to focal cerebral ischemia.

As a consequence of ischemia, microglial numbers decrease in the ipsilateral hemisphere after experimental stroke whereas leukocyte infiltration increases at the same time^4^,^38^. Stroke is known to stimulate bone marrow production of myeloid cells, including monocytes and granulocytes, which are subsequently recruited to the brain^38^,^39^. Also T cell numbers in the bone marrow have been shown to increase after experimetal stroke^39^. We therefore investigated changes in myeloid cell populations in the bone marrow from LysMcreTNF^fl/fl^ and TNF^fl/fl^ mice after pMCAO. We found that numbers and percentages of CD11b^+^CD45^+^Gr1^−^ monocytes and CD45^+^CD3^+^ T cells were significantly increased in LysMcreTNF^fl/fl^ mice 24 hours after pMCAO suggesting that ablation of mesodermally-derived TNF results in an increase in the number of newly produced, bone marrow-derived monocytes and T cells. The signals that trigger leukocyte mobilization from the bone marrow after stroke are unknown and may represent potential targets for future therapies^40^,^41^. The bone marrow indirectly receives central autonomic innervation involving forebrain areas such as the insular and piriform cortex^42^, areas which often are affected by ischemia. This may account for the increased mobilization of monocytes and T cells in LysMcreTNF^fl/fl^ mice, which also display significantly increased infarcts. We and others have shown that following cerebral ischemia cellularity decreases in the spleen^3^,^43^,^44^, which can be attributed to increased apoptotic cell death of lymphocyte populations^43^. Therefore, it is possible that the increase in CD3^+^ T cells in LysMcreTNF^fl/fl^ mice compared to TNF^fl/fl^ mice can be due to decreased ischemia-induced apoptosis of spleen lymphocytes. On the other hand, early splenic responses after stroke involve increased production of pro-inflammatory mediators^45^. It is therefore possible that the pro-inflammatory enviroment is altered in such a way that less T cells are deployed into the blood.

We have previously shown that the number of infiltrating leukocytes is significantly increased in TNF^−/−^ compared to WT mice 24 hours after stroke^4^. Since CD11b expression levels, known to be involved in inflammation by regulating leukocyte adhesion and migration^46^, were increased in naïve microglia, we investigated whether myeloid-derived TNF affected leukocyte infiltration into the ischemic brain. We found that infiltration of macrophages, granulocytes and T cells was not affected in LysMcreTNF^fl/fl^ mice at this time point, nor at 6 hours after pMCAO. Anti-TNF therapies decrease the number of granulocytes infiltrating the ischemic infarct 24 hours after pMCAO^3^, an effect suggested to be mediated via the acute phase response in the liver^47^. Since TNF is not deleted from liver cells in LysMcreTNF^fl/fl^ mice^26^, it is possible that signals mediating granulocyte influx into the brain are not altered in these mice. Suprisingly, macrophage infiltration was not affected in LysMcreTNF^fl/fl^ mice, given that we previously demonstrated reduced infiltration of macrophages into the brain of mTNF^Δ/Δ^ mice 24 hours after pMCAO^5^. As monocytes/macrophages have been shown to infiltrate the ischemic brain relatively late after pMCAO^48^, it is possible that the time point investigated in the present study is not optimal for addressing the influx of macrophages. Nevertheless, we have previously shown, that only microglial-derived TNF, and not macrophage-derived TNF, plays a role in the neuroprotective effects of TNF after pMCAO^4^, which was also supported by this study where we found no TNF^+^ leukocytes 6 hours after pMCAO.

All together, the present study supports microglial-derived TNF as beneficial and neuroprotective in the acute phase after experimental stroke and as a strong modulator of the neuroinflammatory response at later time points after experimental ischemia.

## Materials and Methods

### Mice

MLys1^cre/^x TNF^flox/flox^ (LysMcreTNF^fl/fl^) breeding couples, with a specific deletion of TNF in myeloid cells^26^, were transferred from the Russian Academy of Sciences, Moscow to the Biomedical Laboratory at the University of Southern Denmark where they were established as a colony. The extent of TNF gene deletion in macrophages and neutrophils is almost complete (<98%), with no deletion in liver and thymus^26^. Littermate TNF^flox/flox^ (TNF^fl/fl^) mice, with normal TNF epxression^26^, were used as controls. In addition, MLys1^cre/^mice^49^ were purchased from the Jackson Laboratory and transferred to the Miami Project to Cure Paralysis where they were crossed with Rosa26-loxp-Stop-loxp-tdTomato reporter mice (established colony)^50^ to generate LysM^tdTomato^ mice^51^. All mice used in the present study were adult (7–10 weeks) males. All experimental protocols were approved by The Danish Veterinary and Food Administration (J. No. 2011/561–1950 and 2013-15-2934-00924) and according to the University of Miami IACUC and NIH guidelines. All efforts were made to minimize pain and distress and the methods were carried out in accordance with the relevant guidelines.

### Genotyping

TNF^fl/fl^ mice were genotyped by a two-oligonucleotide primer PCR assay and Cre-mediated recombination and completion of deletion were established using a three-oligonucleotide primer PCR assay^26^. Primer sequences were: KO41 (5′-TGAGTCTGTCTTAACTAACC) and KO42 (5′-CCCTTCATTCTCAAGGCACA) for TNF and a combination of primers Cre8 (CCCAGAAATGCCAGATTACG), Mlys1 (CTTGGGCTGCCAGAATTTCTC) and Mlys2 (TTACAGTCGGCCAGGCTGAC) for Cre.

### Cerebrovascular anatomy

The circle of Willis and its branches were examined in LysMcreTNF^fl/fl^ and TNF^fl/fl^ mice essentially as previously described^4^ using a Toluidine blue (TB) solution. Vessels were photographed under a dissecting microscope and the left an right posterior communicating (PCOM) arteries scored as follows: 0, absent; 1, present, but poorly developed (hypoplastic); 2, well formed. A single PCOM score was obtained by averaging left and right scores.

### Body Composition Measurements

Total tissue mass (g), fat mass (g), fat-%, lean tissue mass (g), lean-%, bone area (cm^2^), bone mineral content (BMC, g/cm) and bone mineral density (BMD, g/cm^2^) were measured in naïve LysMcreTNF^fl/fl^ and TNF^fl/fl^ using dual-energy X-ray absorptiometry (DXA) using a PIXImus2 (Version 1.44; Lunar Corporation, Madision, WI, USA) as previously described^5^,^52^.

### Induction of focal cerebral ischemia

The distal part of the left middle cerebral artery was permanently occluded (pMCAO) under Hypnorm/Stesolid anesthesia [Fentanyl Citrate (0.315 mg/mL; Jansen-Cilag) and Fluanisone (10 mg/mL; Jansen-Cilag); and Diazepamum (5 mg/mL; Dumex)]^22^,^28^. After surgery, mice were supplied with 0.9% saline and maintained in a 28 °C controlled environment. Mice with 5 days survival were housed in the behavioral room in the conventional animal facility after 24 hours. For post-surgical analgesia, mice were treated with Temgesic (0.001 mg/20 g body weight buprenorphium; Reckitt and Colman) three times at 8 hours intervals starting prior to surgery.

### Positron emission tomography (PET) analysis

Micro PET imaging was performed on a Siemens Inveon PET scanner. The animals were scanned twice. The first scan was performed in the acute phase, 1–2 hours after surgery, and the second scan 24 hours later. Animals were placed in prone position on a heated scanner bed and injected with ^18^[F]-FDG via a tail vein catheter. The animals were anesthetized throughout the entire imaging procedure using a mixture of 1.5–2% isoflurane and 100% oxygen.

At day 0 (1–2 hours after pMCAo) 10.9 ± 2.4 MBq ^18^[F]-FDG was injected and data was acquired for 90 min. Data was binned in 20 frames (4 × 30 s + 10 × 1 min + 1 × 3 min + 5 × 15 min), beginning with short frames to capture the fast vascular phase moving to a slower frame rate appropriate for the dynamics beyond 15 min. At day 1 (24 hours after pMCAo) 10.7 ± 1.2 MBq ^18^[F]-FDG was injected. After a 30 min delay a 15 min static image was acquired.

All images were reconstructed using OSEM3D/MAP matrix 128 × 128, 4 OSEM3D iterations and 18 MAP iterations with requested resolution of 0.8 mm. A zoom factor of 2 was applied yielding a voxel size of approximately 0.4 mm × 0.4 mm in the XY-plane and a slice thickness of 0.8 mm. The reconstruction was carried out with the build in algorithm of Siemens Inveon Acquisition Workplace software version 1.5.

A computer tomography (CT) image of a single animal was acquired and used as an anatomical reference. All images were analyzed and the kinetic modeling done within the Siemens Inveon Research Workplace software version 4.2. The reference CT and the PET images were manually co-registered. The images acquired at day 0 were analyzed using a 2-tissue-compartment irreversible FDG model. Image derived blood input functions^53^ were used to calculate the 3 rate constants K_1_ (transport rate constant from blood to brain tissue), k_2_ (rate constant from brain tissue back to blood), k_3_ (binding in the cells (phosphorylation))^31^. The FDG uptake (influx) rate constant K_i_ was calculated as K_i_ = K_1_k_3_/(k_2_ + k_3_). The glucose uptake (metabolism) is proportional to K_i_, glucose uptake = K_i_ (blood glucose/LC), where LC is the lumped constant. The FDG uptake is a very sensitive measure and is influenced by many factors including dietary condition (insulin level), ambient temperature and mode of anesthesia^54^. In order to overcome some of these limitations, we used the animals as their own reference and calculated the rate constants from the ipsilateral volume of interest (VOI) normalized to contralateral reference rate constants. Curves are presented as standardized uptake values (SUV = tissue activity concentration (Bq/mL) x Animal weight (g)/injected activity (Bq)).

Volumes of interest were semi-automatically made by first drawing a volume covering the increased FDG uptake in the ipsilateral region. The final VOI was calculated by setting a threshold of 70% of maximum within the last image of the dynamic series. This VOI was mirrored onto the contralateral brain hemisphere as a reference VOI for the unaffected brain tissue. The blood input VOI was manually created in the first or second frame where the vena cava was clearly visualized. The blood input VOI was manually drawn as the part of vena cava with highest activity.

### Behavioral Tests

#### Open field test

Locomotor activity was investigated using the open field test in a group of naïve LysMcreTNF^fl/fl^ and TNF^fl/fl^ mice as previously described^52^ in order to detect potential phenotypic differences in locomotor- and anxiety-related activities. In addition, locomotor activity was investigated 2 days after pMCAO in order to test for changes in behavior induced by the ischemic brain lesion. The total distance traveled (cm), speed (V(mean), cm/sec), center/perimeter ratio, and entries into the three zones (wall, inter peri and center of the box) were recorded automatically for 10 min. Rearing, time to first rear, grooming and droppings were recorded manually and are given as number of events (n).

#### Y-maze spontaneous alternation test

In order to test spatial leaning and memory in naïve LysMcreTNF^fl/fl^ and TNF^fl/fl^ the spontaneous alternation test was performed using a Y-maze as previously described^52^. Each mouse was placed in the arm designated (A) of the Y-maze and allowed to freely explore the maze for 8 min. The number of entries, except for the first two, and the number of triads were recorded and the percentage of alternation was calculated.

#### Rotarod test

In order to evaluate motor coordination/performance and balance^52^, the rotarod test (LE8200, Panlab Harvard Apparatus) was performed. The test comprised a pre-training part prior to surgery (30 sec at 4 rotations per min (rpm)) and a trial part consisting of 4 trials (T1 – T4, with at least 15 min in-between) 5 days after surgery. Mice were placed on the rotarod set in accelerating mode. Speed of the rotor was accelerated from 4 to 40 rpm over 5 min. Total time spent on the rotarod for each mouse was measured.

#### Rung walk test

In order to test stepping, inter-limb coordination and balance, the rung walk test was performed 2 days after pMCAO as previously described^6^,^55^. The total number of mistakes on each front and hind paw was plotted for analysis of asymmetry. Prior to surgery, mice were pre-trained in the rung walk test and no asymmetry was observed under baseline conditions (data not shown).

#### Grip strength test

The grip strength measuring device (BIO-GT-3, BIOSEB) was used to study neuromuscular function in mice subjected to pMCAO. The peak amount of force was recorded in 5 sequential trials and the highest grip value was recorded as the score^4^. We analyzed the grip strength in individual (left and right) front paws prior to (baseline) and 3 and 5 days after pMCAO. The unit of force measured is presented as grams (g). Asymmetry between paws in individual mice following pMCAO was calculated and is presented as delta (Δ) grip strength^5^.

### Tissue processing

#### Fresh frozen tissue

Mice were killed by cervical dislocation, and brains quickly removed, frozen in CO_2_ and cut coronally in six parallel series of 30 μm and stored at −80 °C until further processing^5^. Blood samples were collected in EDTA-coated Eppendorf tubes, spun 2 × 10 min at 3,000 g, 4 °C, and plasma stored at −80 °C until further processing.

#### Paraformaldehyde (PFA) fixed tissue

LysM^tdTomato^ mice were anesthetized with Ketamine/Xylazine (100 mg/10 mg/kg) and transcardially perfused with 4% PFA. For cell counting, tissue was processed as previously described^51^, and brains were cut into 20 μm-thick, sagittal cryostat sections and stored at −20 °C until further processing. For cortical volume estimations, LysMcreTNF^fl/fl^ and TNF^fl/fl^ mice were overdosed using Pentobarbital (200 mg/ml)/Lidocainhydrochlorid (20 mg/ml) and transcardially perfused with 4% PFA. Brains were fixed in 4% PFA overnight, changed to 1% PFA and finally 0.1% PFA before they were horizontally vibratome-cut into six 60 μm-thick parallel series of free floating sections and cryoprotected in de Olmo’s solution^5^. For immunofluorescent staining, brains were cryoprotected in 20% sucrose overnight, frozen in CO_2_ snow and cut into 10 series of parallel 20 μm-thick coronal sections on a cryostat. Sections were stored at −20 °C until further processing.

### Volume estimations

#### Infarct volume

Every sixth section (1 series) from mice with 24 hours and 5 days survival after pMCAO was stained with TB for estimation of infarct volume using the Cavalieri principle^28^,^56^. The rostrocaudal distribution of the infarct was analyzed as previously described^5^ using the anterior commisure as an antomical landmark.

#### Neocortical volume

The volume of the neocortex was estimated in naïve TNF^fl/fl^ and LysMcreTNF^fl/fl^ mice using Cavalieri’s principle as previously decribed in detail^5^.

### Immunostaining

#### Immunohistochemistry

In fresh-frozen sections, TNF was detected using a rabbit anti-TNF antibody (clone R4-6A2, 1:200, Pierce Antibody Products) and AP-conjugated anti-rabbit antibody as previously described^27^. Primary microglia and primary astrocytes were stained for microglial CD11b (clone M1/70.15, 1:600, Abdserotec) and astroglial GFAP (1:4,000, Dako) in order to evaluate the purity of the cultures. Substitution of the primary antibody with rabbit IgG (DakoCytomation) or IgG1 gave no signal.

#### Immunofluorescent staining

Immunofluorescent staining for TNF co-locatization was performed essentially as described in Clausen *et al*.^57^ using rabbit anti-TNF, rat anti-CD11b and mouse anti-human GFAP-Alexa Fluor-488 (clone 131-17719, 1:400, Life Technologies) antibodies. Donkey anti-rabbit Alexa Fluor-594 and goat anti-rat Alexa Fluor-488 antibodies (1:200, Invitrogen) were used as secondary antibodies. Rabbit IgG was used as a serum control, rat IgG2_b_ (IG-851125, Biosite) and mouse IgG-488 (Invitrogen) were used as isotype controls. Substitution of the primary antibody with isotype control or rabbit IgG gave no signal. Digital images were captured using an Olympus BX51 microscope connected to a PC containing DP manager software (version 2.1.1.158). Figures were composed using Photoshop CS5 software.

LysMcre co-localization in the adult brain was investigated using rat anti-CD11b (clone M1/70.15, 1:500, Invitrogen), rat anti-GFAP (clone 2.2B10, 1:2,000, Invitrogen), mouse anti-NeuN (clone A60, 1:200, Millipore), rabbit anti-red fluorescent protein (RFP) (1:4,000, Rockland), and rabbit anti-Olig2 (1:200, Millipore). Donkey anti-mouse Alexa Fluor-488, donkey anti-rat Alexa Fluor-488, donkey anti-rabbit Alexa Fluor-488, and goat anti-rabbit Alexa Fluor-568 (1:500) were used as secondary antibodies. DAPI (4’,6-diamidino-2-phenylindole) was used as a nuclear marker. Sections were coverslipped using Vectashield (Vectorlabs). Digital images of the cortex were captured using a Nikon Eclipse Ti epifluorescent microscope with NIS Elements software.

### Estimation of cell numbers

#### Cell counting

Quantification of LysM^+^ cells was based on co-localization of the tdTomato reporter signal, which was enhanced using a RFP antibody with the various cell identification markers (NeuN, CD11b, and GFAP). Co-localization with oligodendroglial Olig2 was performed with the endogenous tdTomato signal. In total, 151–171 LysM^+^ (tdTomato) cells spread across at least three non-consecutive tissue sections were counted per animal (n = 3). All cells had a clearly identifiable DAPI^+^ nucleus.

### Primary microglia and astrocyte cultures

For preparation of primary microglia and astrocyte cultures, 0–2 day old LysMcreTNF^fl/fl^ and TNF^fl/fl^ pups were decapitated, the brains isolated, and the meninges removed. Tail biopsies were obtained for genotyping. Brains were then placed in pre-warmed DMEM-GlutaMAX culture media (Gibco) containing 20% FCS and 1% penicillin/streptomycin (Gibco), mechanically homogenized by pipetting and filtered through a 70 μm cell strainer (Falcon). Cells corresponding to the number obtained from 1 brain were seeded into poly L-ornithine pre-coated 24-well culture dishes (Costar) and grown at 36 °C (20% O_2_ and 5% CO_2_) for up to 21 days. Fifty percent of the culture medium was changed every 3–4 days. After 7 days, the medium was changed to culture medium containing 10% FCS and 1% penicillin/streptomycin and cells grown for an additional 7 days. After 14 days *in vitro*, microglia were removed from the layer of confluent astrocytes by gently shaking at 100 rpm for 2 hours at room temperatur. Microglia were harvested from the same dish twice with 7 days intervals. Microglia and astrocytes were separated and centrifuged separately at 1,000× g for 5 min at room temperature. The supernatant was removed and cells resuspended in medium before plated into 24-well plates (20,000 cells/well). Microglia were then stimulated with 10 ng/mL lipopolysaccharide (LPS) (from Escherichia coli 0111:B4, Sigma) and astrocytes with either 10 ng/ml recombinant TNF (Sigma) or 10 ng/ml recombinant interleukin (IL)-1β (Sigma) for 24 hours. Unstimulated cultures were used as controls. After 24 hours, the media was removed and stored at −80 °C until further analysis. Cells were then prepared for immunohistochemistry by 3 x rinses in 0.15 M Sorensen Buffer (SB), fixation for 20 min i 4% PFA, 3 x rinses in 0.15 M SB and stored in 0.15 M SB for evaluation of purity (described above), which was found to be >95% (data not shown).

### ELISA for TNF

The amount of TNF present in the media from LPS-stimulated and unstimulated microglia and TNF- or IL-1β-stimulated or unstimulated astrocyte cultures was examined using the Mouse TNF ELISA MAX^TM^ Deluxe Sets (Biolegend) according to the manufacturer’s instructions. The obtained data was processed and analyzed by the interface software program SoftMax^®^Pro. Each standard and test was determined in duplicate.

### Flow cytometry

#### Cre expression in the cortex of LysM^tdTomato^ mice

For flow cytometry, cells from LysM^tdTomato^ mice were isolated as previously described^58^, with minor modifications. Briefly, after transcardial perfusion with PBS, cortices were dissected out and placed in cold Hanks’ Balanced Salt Solution (HBSS). Tissues were enzymatically dissociated using the neural tissue dissociation kit containing papain (Miltenyi Biotec) and washed in cold HBSS. Suspensions were cleared from myelin by incubation with Myelin Removal Beads II followed by negative selection with LS columns (Miltenyi Biotech), according to manufacturer’s instructions. Cells were washed in FACS buffer (eBioscience), blocked with anti-CD16/32 (FcR block, eBioscience) to prevent non-specific staining, and stained with FITC-anti-CD45 (1:200, eBioscience) and PE-Cy7-anti-CD11b (1:200, eBioscience). Cell suspensions were fixed in 1% PFA and resuspended in FACS buffer. Samples were analyzed with an LSRII flow cytometer (BD Biosciences) equipped with FACS-Diva 6.0 software (BD Biosciences).

#### Cell population analysis in TNF^fl/fl^ and LysMcreTNF^fl/fl^ mice

Flow cytometry was performed as previously described using the FACSVerse (BD Biosciences) and data analyzed using the FACSuite software^3^,^5^. Microglia [CD11b^+^CD45^dim^ or CD11b^+^CD45^dim^Ly6C^low^Ly6G^−^], macrophages [CD11b^+^CD45^high^Gr1^−^ or CD11b^+^CD45^high^Ly6C^high^Ly6G^−^], granulocytes [CD11b^+^CD45^high^Gr1^+^ or CD11b^+^CD45^high^Ly6C^+^Ly6G^+^], T cells [CD45^high^CD3^+^], TNF^+^ microglia and TNF^+^ macrophages were identified as previously described^3^,^5^.

Prior to fixation, cells were stained for live/dead cells using a Fixable Viability Dye eFluoro 506 (eBioscience) diluted in PBS^5^. For TNF expression analysis, cell suspensions were incubated for 4 to 6 hours *in vitro* with the protein transport inhibitor GolgiPlug (BD Biosciences) and processed as previously described^3^,^4^. A total of 1,000,000 events were collected using FSC and SSC and analysis of the live/dead gate revealed comparable numbers of dead cells in all the samples. Furthermore, blood and spleen samples, in addition to bone marrow at 24 hours after pMCAO, were collected and analyzed for CD45, CD11b, Gr1, and CD3 markers.

Positive staining was determined based on fluorescence levels of the respective isotype and fluorescence minus one (FMO) controls. Antibodies were directly conjugated with fluorochromes: PerCP Cy5.5 anti-CD45 (BD Biosciences, clone 30-F11), PE anti-CD11b (BD Biosciences, clone M1/70), PE-Cy7 anti-Ly6G/Ly6C (Gr1) (Biolegend, clone RB6-8C5), PE-Cy7 anti-Ly6C (BD Biosciences, clone AL-21), BV421 anti-Ly6G (BD Biosciences, clone 1A8), APC anti-TNF (Biolegend, clone MP6-XT22), and APC anti-CD3 (BD Biosciences, clone 145-2C11). Isotype controls used were hamster IgG1κ (BD Biosciences, clone A19-3), rat IgG2b (BD Biosciences, clone A95-1 or Biolegend, clone RTK4530), rat IgG1κ (Biolegend, clone RTK2071), rat IgMκ (BD Biosciences, clone R4-22), Lewis IgG2aκ (BD Biosciences, clone R35-95), and mouse IgG1 (BD Biosciences, clone MOPC-21). The mean fluorescence intensity (MFI) was calculated as the geometric mean of each population in the TNF, CD45 and CD11b positive gates, respectively^3^.

### Multiplex and Western blotting analyses

#### Protein purification

One series of brain tissue was lysed in 1x Tris Lysis buffer (150 nM NaCl, 20 mM Tris, 1 mM EDTA, 1 mM EGTA, 1% Triton-x100, pH 7.5) containing phosphatase inhibitor (Sigma) and cOmplete mini, EDTA-free proteinase inhibitor cocktail (Roche). Protein estimations were performed using the Bradford method.

#### Multiplex analysis

Plasma and brain samples were measured in the MSD Mouse Proinflammatory V-Plex Plus Kit (IFNγ, IL-1β, IL-2, IL-4, IL-5, IL-6, IL-10, IL-12p70, CXCL1, TNF; K15012C, Mesoscale) and in brain samples the mouse TNF-RI and TNF-RII Ultra-Sensitive Kits (Mesoscale) using a MSD QuickPlex (SQ120) Plate Reader (Mesoscale) according to the manufacturer’s instructions^5^. Samples were run in duplex and diluted 2- or 4-fold in Diluent 41 prior to measurement. Data was analyzed using MSD Discovery Workbench software. The lower limit of detection was a calculated concentration based on a signal 2.5 SD above the blank (zero) calibrator.

#### Western blot analysis

Western blot analysis for TNF (1:2,000, Abcam) was performed using 20 μg protein extract separated on 4–12% SDS-PAGE gels (Nupage, Invitrogen) essentially as previously described^3^,^4^. As a positive control, 0.5 ng 17 kDa murine recombinant TNF (Sigma Aldrich) was included. Western blotting analysis for SAPK/JNK (Cell Signaling, 1:1,000), phosphorylated (p)-SAPK/JNK (Thr183/Tyr185) (Cell Signaling, 1:1,000), p44/p42 MAPK (Cell Signaling, 1:1,000), p-p44/p-p42 MAPK (ERK1/2)(Cell Signaling, 1:1,000), p-38 (Cell Signaling, 1:1,000), p-p38 MAPK (Tyr180/Tyr182)(Cell Signaling, 1:1,000), and Iba1 (Wako, 1:500) was performed by resolving equal amounts of protein lysates by SDS-PAGE on NuPage Bis 4–12% gels, using MOPS SDS (Invitrogen) containing 0.25% antioxidant (Invitrogen) essentially as previously described^6^,^59^. TFIIB (1:1,000, Cell Signaling), GAPDH (1:2,500, Abcam) and α-actin (1:8,000, Millipore) were used as loading controls. SeeBlue Plus2 prestained standard (Invitrogen) was used as a molecular weight marker. Bands were quantified with Quantity One software (Biorad). Analysis was performed on 2–4 independent gels with n = 2–3/group and data were normalized to TFIIB, GAPDH or α-actin and represented as percentages relative to naïve TNF^fl/fl^ mice or as ratios (for MAPK).

### Human stroke tissue

The study was performed on *post mortem* brain tissue from two stroke cases obtained from the Department of Pathology, Odense University Hospital and the use of human brains was approved by the Danish Biomedical Research Ethical committee for the Region of Southern Denmark (permission number S-20080042). Sex, age, infarcted brain area and age of infarct are given in Suppl. Table 4. Specimens from these brains have been part of a previous study on surfactant protein-D in human ischemic brain tissue^60^.

### Immunohistochemistry in human stroke tissue

Tissue blocks containing infarcted human brain tissue were imbedded in paraffin and 2 μm thick, serial sections were cut on a microtome, deparaffinized, blocked in 1.5% H_2_O_2_ in Tris-buffered saline (TBS) and demasked in T-EG buffer (10 mM Tris + 0.5 mM EGTA, pH 9.0), whereafter they were loaded onto a Dako autostainer (Dako, Denmark)^61^. Sections were stained using the following primary antibodies: rabbit anti-Iba1 (ionized calcium binding adaptor molecule 1, 1:1,000, Wako), mouse anti-CD45 (1:200, Dako), mouse anti-CD68 (1:100, Dako), rabbit anti-GFAP (glial fibrillary acidic protein, 1:2,000, Dako), rabbit anti-TNF (1:100, Endogen)^4^,^23^,^27^, rabbit anti-TNFR1 (1:50 (H-271), Santa Cruz), and rabbit anti-TNFR2 (1:50, Sigma-Aldrich). Secondary antibodies included EnVision + System horseradish peroxidase (HRP)-labelled Polymer (Dako) for CD45, CD68 and GFAP, Advance HRP (Dako) for TNF and TNFR1, and PowerVision + Poly-HRP IHC detection system (AH Diagnostics) for TNFR2. Omission of primary antibody and comparable concentrations of rabbit IgG were used to control for unspecific binding in the immunohistochemical protocols for TNF, TNFR1 and TNFR2 and these sections were devoid of staining. In addition, parallel sections were stained for hematoxylin and eosin (HE) using standard protocols at the Department of Pathology, Odense University Hospital.

### Statistics

For multiple comparisons, one-way or two-way ANOVA was applied. When appropriate, repeated measures were used. Each test was followed by the relevant post-hoc test. For single comparisons unpaired and paired student’s t-tests were applied. P values ≤ 0.05 were considered statistically significant. Data are presented as mean ± SEM.

## Additional Information

**How to cite this article**: Clausen, B. H. *et al*. Conditional ablation of myeloid TNF increases lesion volume after experimental stroke in mice, possibly via altered ERK1/2 signaling. *Sci. Rep.* **6**, 29291; doi: 10.1038/srep29291 (2016).

## Supplementary Material

Supplementary Information

## Figures and Tables

**Table 1:** K_*i*_, influx rate for an irreversible model; K_*1*_, rate constant from blood to tissue; K_*2*_, efflux rate from tissue back into blood; K_*3*_, rate of binding.

	**TNF**^**fl/fl**^	**LysMcreTNF**^**fl/fl**^	**P value**
K_i_ (normalized)	1.63 ± 0.09	2.24 ± 0.23	*P = 0.03
K_1_ (normalized)	0.46 ± 0.06	0.66 ± 0.05	*P = 0.03
k_2_ (normalized)	0.30 ± 0.05	0.53 ± 0.06	*P = 0.02
k_3_ (normalized)	1.23 ± 0.15	2.14 ± 0.26	*P = 0.02

All values are normalized to the respectively contralateral rate constants, hence values are relative to contralateral values. Data are presented as mean ± SEM, student’s t test.
