# Supplementary material for: Conditional ablation of myeloid TNF increases lesion volume after experimental stroke in mice, possibly via altered ERK1/2 signaling
Source: Sci Rep. 2016 Jul 7;6:29291. doi: 10.1038/srep29291 (PMC4935869; doi:10.1038/srep29291)
Supplement: Supplementary Information [file srep29291-s1.docx]

**Conditional ablation of myeloid TNF increases lesion volume after experimental stroke in mice, possibly via altered ERK1/2 signaling**

**Online supplement**

**Bettina Hjelm Clausen^1^, Matilda Degn^6^, Mithula Sivasaravanaparan^1^, Torben Fogtmann^1^, Maria Gammelstrup Andersen^1^, Michelle D. Trojanowsky^7^, Han Gao^7^, Svend Hvidsten^4^, Christina Baun^4^, Tomas Deierborg^9^, Bente Finsen^1^, Bjarne Winther Kristensen^2,3^, Sara Thornby Bak^1^, Morten Meyer^1^, Jae Lee^7^, Sergei A. Nedospasov^8^, Roberta Brambilla^7^, Kate Lykke Lambertsen^1,5*^**

*^1^Department of Neurobiology Research, Institute of Molecular Medicine and ^2^Institute of Clinical Research, University of Southern Denmark, Odense, Denmark, ^3^Department of Pathology, ^4^Department of Nuclear Medicine, and ^5^Department of Neurology, Odense University Hospital, Odense, Denmark, ^6^Department of Diagnostics, Rigshospitalet, Glostrup, Denmark, ^7^Miami Project to Cure Paralysis, University of Miami Miller School of Medicine, ^8^Engelhardt Institute of Molecular Biology, Russian Academy of Sciences and Lomonsov Moscow State University, Russia, ^9^Department of Experimental Medical Sciences, Experimental Neuroinflammation Laboratory, 22100 Lund University, Lund, Sweden,*

**Corresponding author:** Kate Lykke Lambertsen, klambertsen@health.sdu.dk, J.B. Winsloewsvej 21 St., DK 5000, Odense, DK; e-mail klambertsen@health.sdu.dk, tel. +4565503806

**Supplemental Figure 1**

| **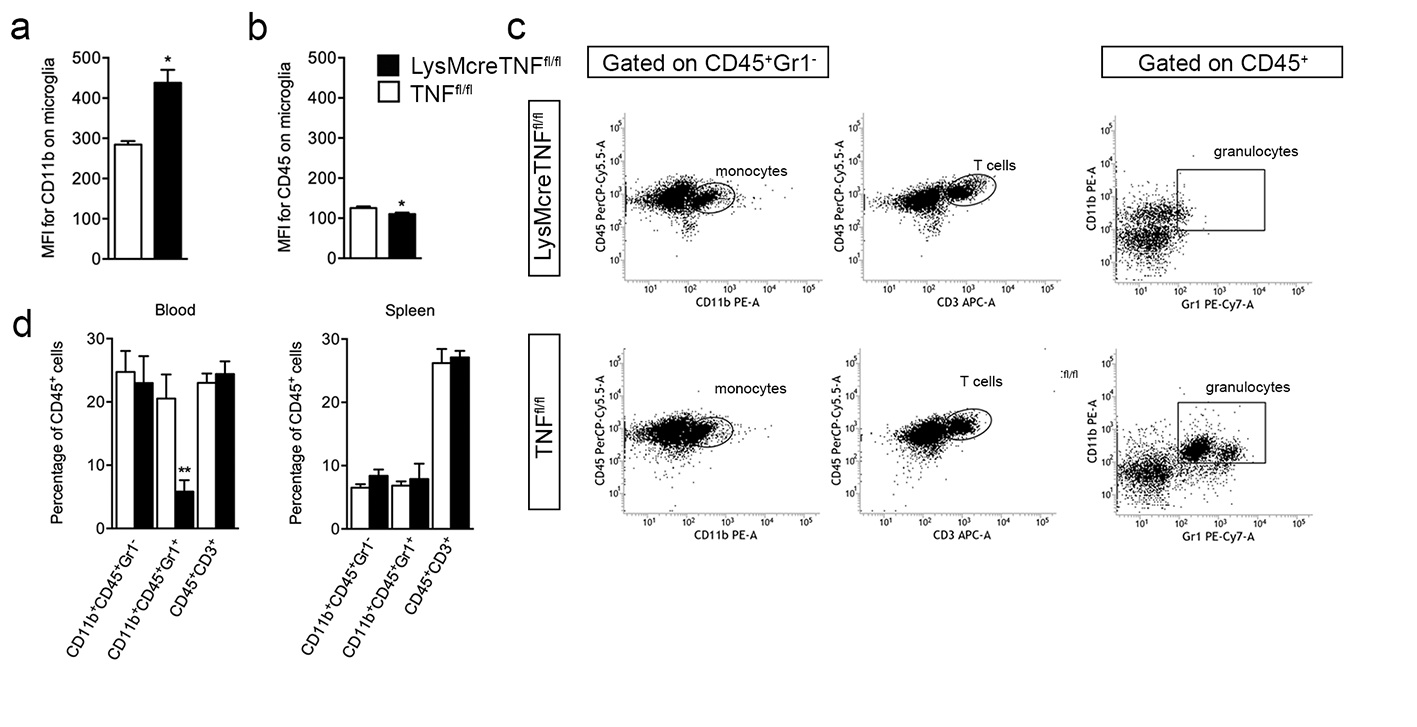** |
| --- |

**Supplemental Figure 1. Flow cytometric analysis of monocyte, granulocyte and T cell populations in blood and spleen from naïve LysMcreTNF^fl/fl^ and TNF^fl/fl^ mice.** (**a,b**) Mean fluorescence intensity (MFI) expression levels for CD11b (a) and CD45 (b) on naïve microglia from TNF^fl/fl^ and LysMcreTNF^fl/fl^ mice (n = 5 mice/group, t-test). (**c**) Gating strategy for monocytes, granulocytes and T cells in blood and spleen from naïve TNF^fl/fl^ and LysMcreTNF^fl/fl^ mice (shown for blood samples). FSC/SSC was used to define a gate comprising leukocytes and lymphocytes. Next, at gate was defined to include only live cells in the further analysis. Monocytes were identified as CD11b^+^CD45^+^Gr1^-^ cells, granulocytes as CD11b^+^CD45^+^Gr1^+^ cells and T cells as CD45^+^CD3^+^ cells. (**d**) Flow cytometric analysis of blood samples revealed that the percentage of granulocytes in the blood was significantly reduced in LysMcreTNF^fl/fl^ compared to TNF^fl/fl^ mice. Similar percentages of monocytes and T cells were found between TNF^fl/fl^ and LysMcreTNF^fl/fl^ mice (n = 6-9/group, t-test). In the spleen, the percentages of monocytes, granulocytes and T cells were found to be comparable between TNF^fl/fl^ and LysMcreTNF^fl/fl^ mice (n = 6-9/group, t-test). *P≤0.05. All data are presented as mean ± SEM.

**Supplemental Figure 2**

| **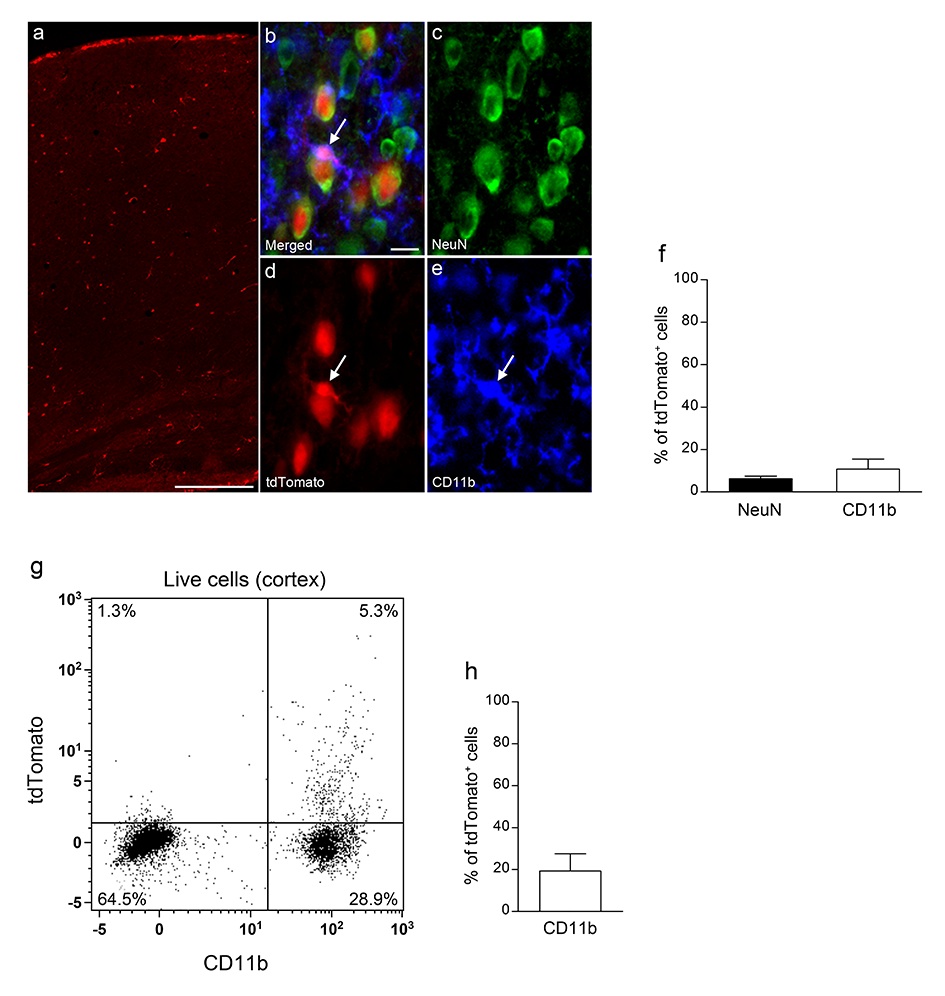** |
| --- |

**Supplemental Figure 2. Characterization of LysMcre transgene expression under naïve conditions.** (**a**) Cre-mediated recombination in dtTomato^+^ cells (red) was observed throughout the neocortex and was found to have taken place both in NeuN^+^ neurons (green) and CD11b^+^ microglia (blue). (b) Total tdTomato^+^ cells comprised approximately 12% of all CD11b^+^ microglia and 5% of all neurons (n = 3). Scale bars: Low magnification = 250 μm and high magnifications = 10 μm. (**c**) Flow cytometry analysis revealed that tdTomato^+^ cells comprised approximately 20% of all CD11b^+^ cells (n = 3). All data are presented as mean ± SEM.

**Supplemental Figure 3**

| **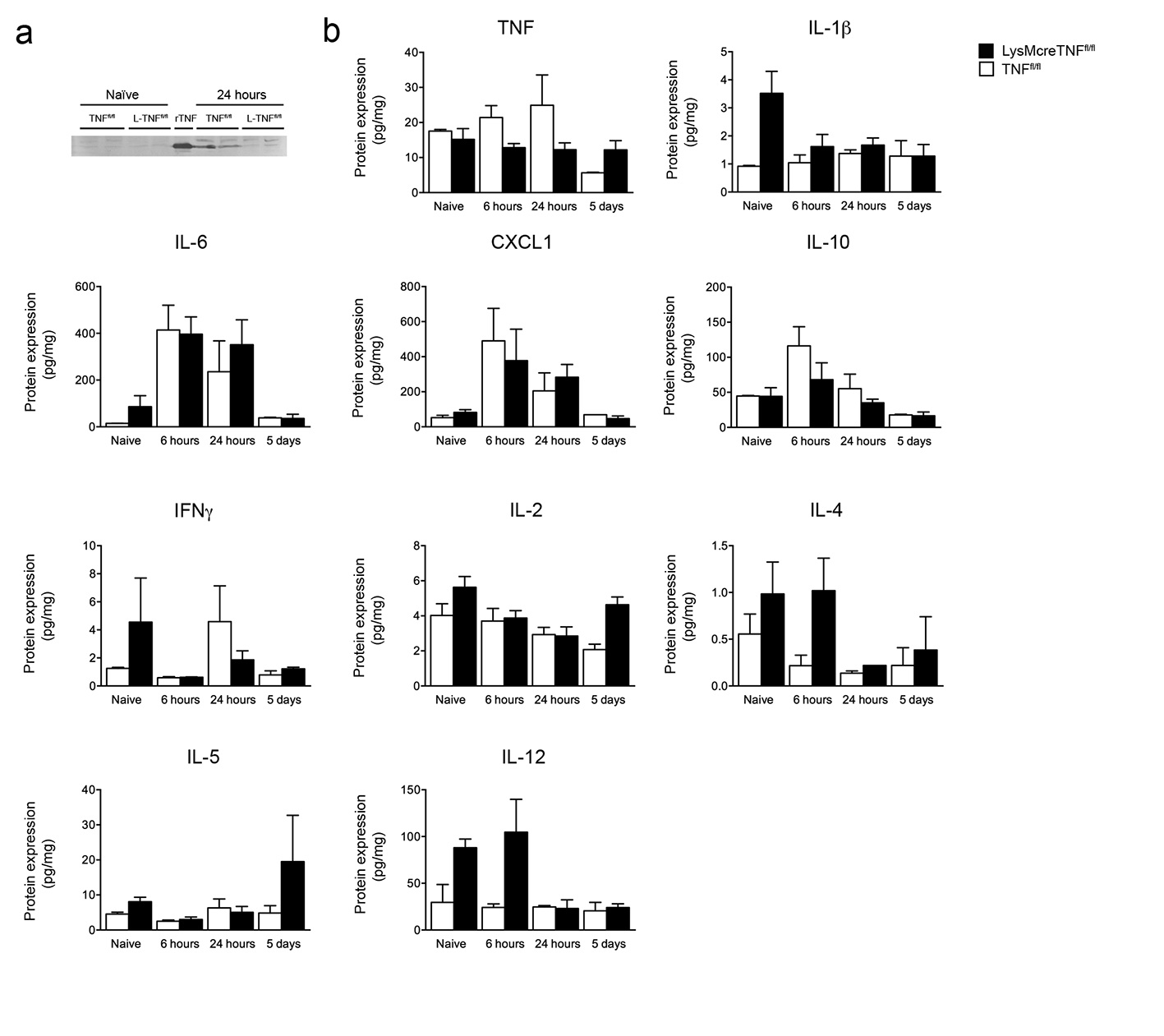** |
| --- |

**Supplemental Figure 3. Inflammatory changes after pMCAO.** (**a**) Western blot showing increased TNF expression in TNF^fl/fl^ mice 24 hours after pMCAO as compared to LysMcreTNF^fl/fl^ mice. (**b**) Multiplex analysis of plasma TNF, IL-1β, IL-6, CXCL1, IL-10 IFNγ, IL-2, IL-4, IL-5, and IL-12 protein levels in naïve conditions and 3 or 24 hours, and 5 days after pMCAO (n = 3/group). All data are presented as mean ± SEM.

**Supplemental Figure 4**

| **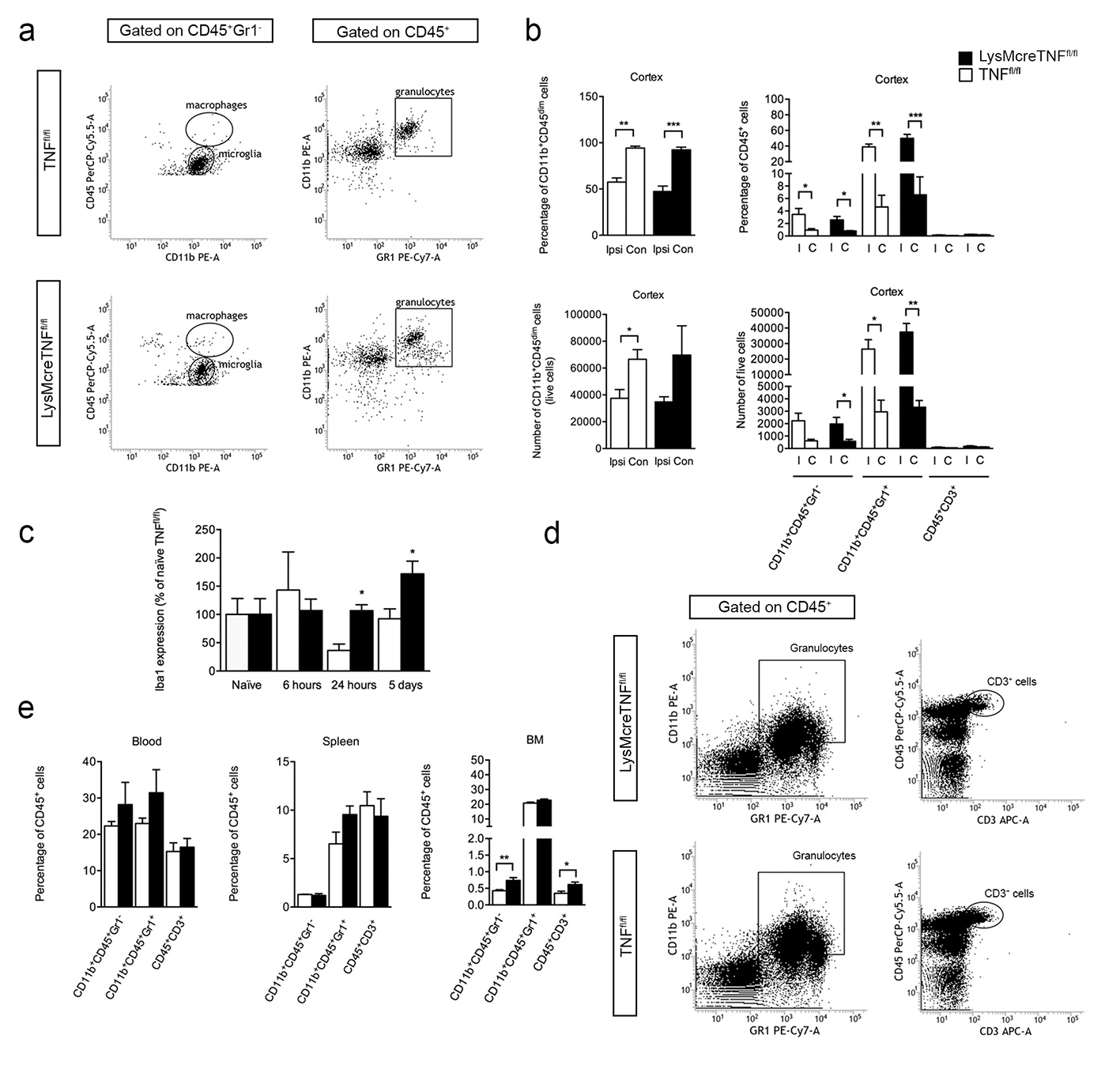** |
| --- |

**Supplemental Figure 4.** **Flow cytometric analysis of monocyte, granulocyte and T cell populations in brain, bone marrow, blood and spleen from TNF^fl/fl^ and LysMcreTNF^fl/fl^ mice 24 hours after pMCAO.** (**a,b**) Gating strategy (a) and flow cytometric analysis of the total number and percentage of CD11b^+^CD45^dim^ microglia, CD11b^+^CD45^high^Gr1^-^ macrophages, CD11b^+^CD45^high^Gr1^+^ granulocytes and CD45^+^CD3^+^ T cells of all cortical CD45^+^ cells in TNF^fl/fl^ and LysMcreTNF^fl/fl^ mice 24 hours after pMCAO (b). Results are expressed as mean ± SEM of 5 animals/groups. (**c**) Quantification of Iba1 protein expression in brain tissue of naïve TNF^fl/fl^ and LysMcreTNF^fl/fl^ mice, 6 hours, 24 hours and 5 days after pMCAO. Data are normalized to α-actin protein expression. Representative experiments are shown. Results, expressed as percentage of naïve TNF^fl/fl^ mice, are mean ± SEM of n = 3/group. (**d**,**e**) Gating strategy (d) and flow cytometric analysis of the percentage of CD11b^+^CD45+Gr1^-^ monocytes, CD11b^+^CD45^+^Gr1^+^ granulocytes and CD45^+^CD3^+^ T cells in blood, spleen and bone marrow (BM) of TNF^fl/fl^ and LysMcreTNF^fl/fl^ mice 24 hours after pMCAO. Results are expressed as mean ± SEM of 5 animals/groups, t-test. *P ≤ 0.05, **P ≤ 0.01.

|  | ***TNF^fl/fl^***  *N Mean SEM* | | | ***LysMcreTNF^fl/fl^***  *N Mean SEM* | | | *Statistics* |
| --- | --- | --- | --- | --- | --- | --- | --- |
| **Locomotor acvitivity (Open field test)**  Horizontal actvity (total distance travelled (m))  Horizontal actvity (speed (cm/sec))  Horizontal activity (zone changes)  Vertical activity (number of rearings) | 16  16  16  16 | 17.95  29.94  106.0  39.06 | 2.09  3.49  18.29  6.44 | 18  18  18  18 | 15.16  25.27  78.17  25.06 | 2.01  3.36  10.76  5.03 | n.s.  n.s.  n.s.  P = 0.09 |
| **Anxiety Test (open field test)**  Grooming  Fecal droppings  Latency to rear (sec)  Center/Peri-meter ratio | 16  16  16  16 | 2.80  3.40  68.53  0.21 | 0.53  0.52  24.79  0.03 | 18  18  18  18 | 1.89  2.39  80.89  0.20 | 0.23  0.35  19.68  0.05 | n.s.  n.s.  n.s.  n.s. |
| **Neuromuscular function**  Grip strength (g)  **Motor function and coordination (rotarod)**  T1 (sec)  T2 (sec)  T3 (sec)  T4 (sec)  Total time on the rod – BL (sec)  **Y-maze test**  SAB-%  Y-maze entries | 6  9  9  9  9  9  16  16 | 151.2  117.8  148.4  152.3  ^§^171.2  589.8  58.92  22.25 | 4.21  20.21  16.76  18.86  17.47  57.29  2.45  1.81 | 6  16  16  16  16  16  19  19 | 157.5  127.9  133.6  147.8  ^*^161.5  526.7  52.63  15.37 | 11.94  15.08  14.62  14.01  13.14  53.83  2.26  1.33 | n.s.  n.s.  n.s.  n.s.  n.s.  n.s.  P = 0.09  **P<0.01 |
| **DXA scan**  Bone mineral density (g/cm^2^)  Bone mineral content (g)  Bone area (cm^2^)  % fat  Total tissue mass (g)  Lean tissue mass (g) | 11  11  11  11  11  11 | 0.049  0.410  8.363  13.97  23.71  20.40 | 0.001  0.019  0.196  0.623  1.031  0.904 | 11  11  11  11  11  11 | 0.049  0.411  8.399  13.27  25.10  21.75 | 0.001  0.015  0.197  0.410  0.806  0.655 | n.s.  n.s.  n.s.  n.s.  n.s.  n.s. |

**Supplemental Table 1. Baseline phenotypic analysis of TNF^fl/fl^ and LysMcreTNF^fl/fl^ mice**

§: P = 0.08 and *P<0.05 between T1 and T4 (Repeated Measures One-way ANOVA)

|  | ***TNF^fl/fl^***  *N Mean SEM* | | | ***LysMcreTNF^fl/fl^***  *N Mean SEM* | | | *Statistics* |
| --- | --- | --- | --- | --- | --- | --- | --- |
| **Locomotor acvitivity (Open field test)**  Horizontal actvity (total distance travelled (m))  Horizontal actvity (speed (cm/sec))  Horizontal activity (zone changes)  Vertical activity (number of rearings) | 18  18  18  18 | 18.24  31.90  63.72  15.39 | 1.49  2.56  9.22  2.03 | 20  20  20  20 | 16.26  27.15  56.60  18.55 | 1.27  2.13  7.34  2.49 | n.s.  n.s.  n.s.  n.s. |
| **Anxiety Test (open field test)**  Grooming  Fecal droppings  Latency to rear (sec)  Center/Peri-meter ratio | 18  18  18  18 | 5.72  4.28  105.3  0.06 | 0.10  0.47  29.6  0.01 | 20  20  20  20 | 3.65  4.40  121.0  0.09 | 0.37  0.47  19.7  0.03 | P = 0.05  n.s.  n.s.  n.s. |
| **Weight (g)**  BL  D3  D5 | 15  15  15 | 27.07  ^§^24.73  ^§^25.25 | 0.86  0.75  0.68 | 25  25  25 | 25.64  ^§^23.96  ^§^24.57 | 0.42  0.39  0.33 | n.s.  n.s.  n.s. |

**Supplemental Table 2. Behavioral analysis after focal cerebral ischemia.**

§: Compared to BL ***P<0.001 (Repeated One-way ANOVA)

|  | ***TNF^fl/fl^***  *N Mean SEM* | | | ***LysMcreTNF^fl/fl^***  *N Mean SEM* | | |
| --- | --- | --- | --- | --- | --- | --- |
| **TNF (pg/mg)**  6h  5d  **IL-1β (pg/mg)**  6h  5d  **IL-6 (pg/mg)**  6h  5d  **IL-2 (pg/mg)**  6h  5d  **IL-4 (pg/mg)**  6h  5d  **IL-5 (pg/mg)**  6h  5d  **IL-10 (pg/mg)**  6h  5d  **IL-12p70 (pg/mg)**  6h  5d  **IFNγ (pg/mg)**  6h  5d  **CXCL1 (pg/mg)**  6h  5d | 3  5  3  5  3  5  3  5  3  5  3  5  3  5  3  5  3  5  3  5 | 1.03  1.50  0.52  0.68  39.85  27.66  1.08  2.13  0.87  0.37  0.15  0.21  1.62  2.26  29.51  45.39  0.09  0.17  12.15  5.08 | 0.10  0.35  0.17  0.17  13.44  3.53  0.09  0.59  0.31  0.07  0.06  0.08  0.27  0.36  2.77  12.35  0.01  0.04  3.52  0.61 | 3  4  3  4  3  4  3  4  3  4  3  4  3  4  3  4  3  4  3  4 | 1.26  1.10  0.58  0.57  58.33  22.10  1.33  1.31  0.46  0.41  0.07  0.13  0.95  1.43  40.89  35.58  0.12  0.08  12.57  5.04 | 0.73  0.10  0.28  0.09  38.27  3.22  0.71  0.16  0.16  0.11  0.03  0.06  0.43  0.05  25.88  2.14  0.08  0.01  7.11  0.51 |

**Supplemental Table 3. Multiplex analysis of TNF^fl/fl^ and LysMcreTNF^fl/fl^ mice 6 hours and 5 days after pMCAO.**

| **Case** | **Sex/Age of death** | **Infarcted brain area** | **Infarct age** |
| --- | --- | --- | --- |
| #1 | M/57 | Left Internal capsule | > 7 days |
| #2 | M/38 | Right temporal lobe | < 5 days |

**Supplemental Table 4. Clinical data on *post mortem* brain tissue from two stroke cases**
